# A functional analysis of omic network embedding spaces reveals key altered functions in cancer

**DOI:** 10.1093/bioinformatics/btad281

**Published:** 2023-04-21

**Authors:** Sergio Doria-Belenguer, Alexandros Xenos, Gaia Ceddia, Noël Malod-Dognin, Nataša Pržulj

**Affiliations:** Department of Life Science, Barcelona Supercomputing Center (BSC), Barcelona 08034, Spain; Department of Life Science, Barcelona Supercomputing Center (BSC), Barcelona 08034, Spain; Department of Life Science, Barcelona Supercomputing Center (BSC), Barcelona 08034, Spain; Department of Life Science, Barcelona Supercomputing Center (BSC), Barcelona 08034, Spain; Department of Computer Science, University College London, London WC1E 6BT, United Kingdom; Department of Life Science, Barcelona Supercomputing Center (BSC), Barcelona 08034, Spain; Department of Computer Science, University College London, London WC1E 6BT, United Kingdom; ICREA, Pg. Lluis Companys, Barcelona 08010, Spain

## Abstract

**Motivation:**

Advances in omics technologies have revolutionized cancer research by producing massive datasets. Common approaches to deciphering these complex data are by embedding algorithms of molecular interaction networks. These algorithms find a low-dimensional space in which similarities between the network nodes are best preserved. Currently available embedding approaches mine the gene embeddings directly to uncover new cancer-related knowledge. However, these gene-centric approaches produce incomplete knowledge, since they do not account for the functional implications of genomic alterations. We propose a new, function-centric perspective and approach, to complement the knowledge obtained from omic data.

**Results:**

We introduce our Functional Mapping Matrix (FMM) to explore the functional organization of different tissue-specific and species-specific embedding spaces generated by a Non-negative Matrix Tri-Factorization algorithm. Also, we use our FMM to define the optimal dimensionality of these molecular interaction network embedding spaces. For this optimal dimensionality, we compare the FMMs of the most prevalent cancers in human to FMMs of their corresponding control tissues. We find that cancer alters the positions in the embedding space of cancer-related functions, while it keeps the positions of the noncancer-related ones. We exploit this spacial ‘movement’ to predict novel cancer-related functions. Finally, we predict novel cancer-related genes that the currently available methods for gene-centric analyses cannot identify; we validate these predictions by literature curation and retrospective analyses of patient survival data.

**Availability and implementation:**

Data and source code can be accessed at https://github.com/gaiac/FMM.

## 1 Introduction

### 1.1 Network embeddings in cancer research

Cancer is a major public health problem and one of the leading causes of death in the world (Sung et al. 2021). Despite exceptional research efforts, our knowledge about this disease remains incomplete. Meanwhile, the increasing availability of omic biomedical data has yielded an unprecedented opportunity to understand the fundamental mechanisms of cancer. These data are often represented as networks in which nodes are molecular entities, and edges define their relationships, e.g. in protein–protein interaction networks, edges indicate physical interactions between proteins, as measured by biological experiments. To interpret these high-dimensional data, various network-based approaches have been developed (Lotfi Shahreza et al. 2018). In particular, network embedding techniques are considered to be one of the best approaches to decipher these complex biomedical data (Nelson et al. 2019).

Network embedding techniques aim to find a low-dimensional space in which the node closeness in the original network is preserved in the embedding space (Nelson et al. 2019). Defining an optimal number of dimensions of the embedding space is key to properly representing the closeness between the nodes in the space. However, there is no gold-standard approach to find the optimal dimensionality of the embedding space. Thus, researchers have to rely on grid search, domain knowledge, or heuristics (Luo et al. 2021), e.g. the cophenetic correlation coefficient (Brunet et al. 2004) and rule of thumb (Kodinariya and Makwana 2013).

In cancer research, different network embedding algorithms have been used to identify cancer-related genes (Chen et al. 2019), to subtype cancers (Xu et al. 2021), to stratify patients (Gligorijević et al. 2016) and to repurpose drugs (Ceddia et al. 2020). These algorithms include Nature Language Processing-inspired methods, e.g. DeepWalk (Perozzi et al. 2014), and node2vec (Grover and Leskovec 2016), and matrix factorization-based approaches. In particular, Non-negative Matrix Tri-Factorization (NMTF) is an extension of Non-negative Matrix Factorization (NMF) and a well-known machine learning (ML) technique introduced for coclustering and dimensionality reduction (Ding et al. 2006). Unlike NMF, which factorizes the matrix representation of a network into two low-dimensional non-negative matrices, NMTF generates the embedding space by decomposing it into the product of three non-negative matrices, providing more degrees of freedom in the data modeling and analysis than NMF does (Ding et al. 2006). One of the advantages of NMTF over deep neural network-based ML approaches is that it requires way fewer parameters to tune, thanks to the careful modeling of the relationships between the data points that it takes as input. As shown by Xenos et al. (2021), the molecular network embedding space produced by NMTF can have valuable properties, e.g. orthonormality, that may lead to an easier interpretation and deeper scientific insight (Isokääntä et al. 2020).

### 1.2 Problem

Current approaches for mining embedded biological networks use the genes’ embedding vectors as input to machine learning algorithms to perform downstream tasks. These gene-centric approaches have demonstrated their potential in identifying new gene mutations in cancer cells involved in the initiation and progression of the disease (Jin et al. 2019). However, they offer incomplete analyses of cancer data, since they do not take as input the functional implications of such genomic variations. Thus, changing the gene-centric paradigm to a functional-based one could be key to revealing additional functional information about cancer.

### 1.3 Contributions

To improve our understanding of cancer, we generate cancer and control (healthy) gene embedding spaces by applying the NMTF algorithm to the corresponding tissue-specific protein–protein interaction (PPI) networks (detailed below). Then, to explore these gene embedding spaces from a functional perspective, we propose to embed biological functions, represented by Gene Ontology Biological Processes annotations (Bateman et al. 2019), into these gene embedding spaces. Finally, we capture the functional organization of a given gene embedding space with our new Functional Mapping Matrix (FMM), which encodes the mutual positions of the biological function embedding vectors in the space. First, we use our FMM-based method to identify the optimal dimensionality of cancer and control gene embedding spaces. Then, we apply the FMM to explore the functional changes in the most prevalent cancers (breast, prostate, lung, and colorectal) compared with their corresponding control tissues. We find that the changes in the distances between the embedding vectors of biological functions in cancer compared with the control embedding space are related to cancer. Indeed, we observe that cancer changes the distances between embedding vectors of cancer-related biological functions, while it preserves the positions of other biological functions. We exploit this observation to predict novel cancer-related functions, e.g. alternative translational mechanisms, or the response to unfolded protein accumulation. Moreover, we find a set of eight annotations that are altered in all four cancer types. These annotations describe important cellular functions that may be commonly altered in different cancers, e.g. stress-activated MAPK cascade. Also, we demonstrate that our approach is not only restricted to functionally-based analyses of cancer but also can be used to mine for new genomic knowledge from the embedding space. For instance, we use it to identify novel cancer-related genes, i.e. PRDM11, C9orf72, MINDY3, and H4C6, that could have an important role in the studied cancer types. Finally, our method is generic and can easily be applied to any network data with annotated nodes and any embedding space. The application of our FMM goes beyond cancer and can be used to offer a novel perspective on other important open questions in many domains, e.g. finding the optimal dimensionality of an embedding space. Our methodology can be used as a base for developing new data mining algorithms to complement the classic data embedding approaches.

## 2 Materials and methods

### 2.1 Biological datasets

#### 2.1.1 Tissue-specific networks

We analyze cancer and control tissue-specific PPI networks that we generate by using the same methodology as Malod-Dognin et al. (2019). To this end, we collect the experimentally validated PPIs of *Homo sapiens* (human) from BioGRID v.4.2.191 (Oughtred et al. 2019). We model this human PPI data as a PPI network, in which nodes represent genes (or equivalently in this study, their protein products) and edges connect the nodes (genes) whose corresponding proteins physically bind. We use this generic human PPI network to generate our tissue-specific PPI networks. Following Malod-Dognin et al. (2019), we collect the tissue-specific gene expression data for breast, prostate, lung, and colorectal cancer tissues, as well as their corresponding control tissues of origins (breast glandular cells, prostate glandular cells, lung pneumocytes, and colorectal glandular cells, respectively) from the Human Protein Atlas (HPA) database v.20.0 (Pontén et al. 2008). For each tissue, we only consider the genes whose expression value is available in the HPA and that have at least one PPI in the generic human PPI network. We generate our eight tissue-specific PPI networks, in which nodes are genes that are expressed in the corresponding tissue, and two nodes are connected by an edge if they interact in the generic human PPI network. The network statistics of the tissue-specific networks are presented in Supplementary Table S2. In Supplementary Section S2, we also consider species-specific PPI networks whose data collection is described in Supplementary Section S2.1.1.

#### 2.1.2 Network representation

We represent the tissue-specific PPI networks with their positive point-wise mutual information (PPMI) matrices, *X*, where each entry in the matrix contains information about how frequently two nodes co-occur in a random walk in the corresponding PPI network. Following Xenos et al. (2021), we use the DeepWalk closed formula by Perozzi et al. (2014) with its default settings, which uses 10 iterations, to compute the PPMI matrix. This formula can be interpreted as a diffusion process that captures high-order proximities between the nodes in the network; hence, PPMI is a richer representation than the adjacency matrix (Xenos et al. 2021). As a result of the extra information encoded in the PPMI, its corresponding embedding spaces better capture the functional organization of the cell than the ones generated by using the adjacency matrix (the details of this comparison are presented in Supplementary Section S1.2.1).

#### 2.1.3 Biological annotations

We use the Gene Ontology Biological Process (GO BP) annotations of genes’ biological functions in a cell (Bateman et al. 2019). We collected the experimentally validated GO BP annotations of genes from NCBI’s web server (collected on 28 September 2021).

### 2.2 Definition of cancer-related biological annotations

Computational cancer research is usually based on computationally processing information about genes and not their annotations. Although a standard definition of a cancer driver (oncogene) exists (Lee and Muller 2010), there does not exist a standard definition of a cancer-related GO BP term. Oncogenes are a functionally heterogeneous group of genes whose products regulate multiple cellular processes (Pappou and Ahuja 2010). Despite this heterogeneity, oncogenes also participate in common molecular mechanisms that are known to be cancer-related, e.g. cell proliferation (Vicente-Dueñas et al. 2013). Thus, we propose to consider as cancer-related the most representative biological functions in which the oncogenes participate (detailed below).

We download the set of all 725 genes considered to be oncogenes in COSMIC (Forbes et al. 2017) (collected on 01 December 2021). We find the most representative biological functions of these oncogenes by performing an enrichment analysis of our oncogenes set in GO BP functions (based on the hypergeometric test; Rice 2006). A GO BP annotation is considered to be significantly enriched in our set of oncogenes, compared with all other genes, if its enrichment *P*-value is ≤5% after correction for multiple hypothesis testing (Brown 2008). We find 104 significantly enriched GO BP annotations in our set of oncogenes: these are our ‘cancer-related annotations’. To validate our set of cancer-related annotations, we calculate the Lin’s semantic similarity (Lin 1998) between our set of cancer-related functions and the set of 135 ‘cancer hallmark’ annotations defined by Chen et al. (2021). With an average Lin’s semantic similarity between the sets of 0.67, (see Supplementary Fig. S1) we conclude that the two sets are highly functionally related, i.e. our set of cancer-related annotations is related to the cancer hallmarks.

### 2.3 Embedding the protein–protein interaction networks

**Figure 1.**
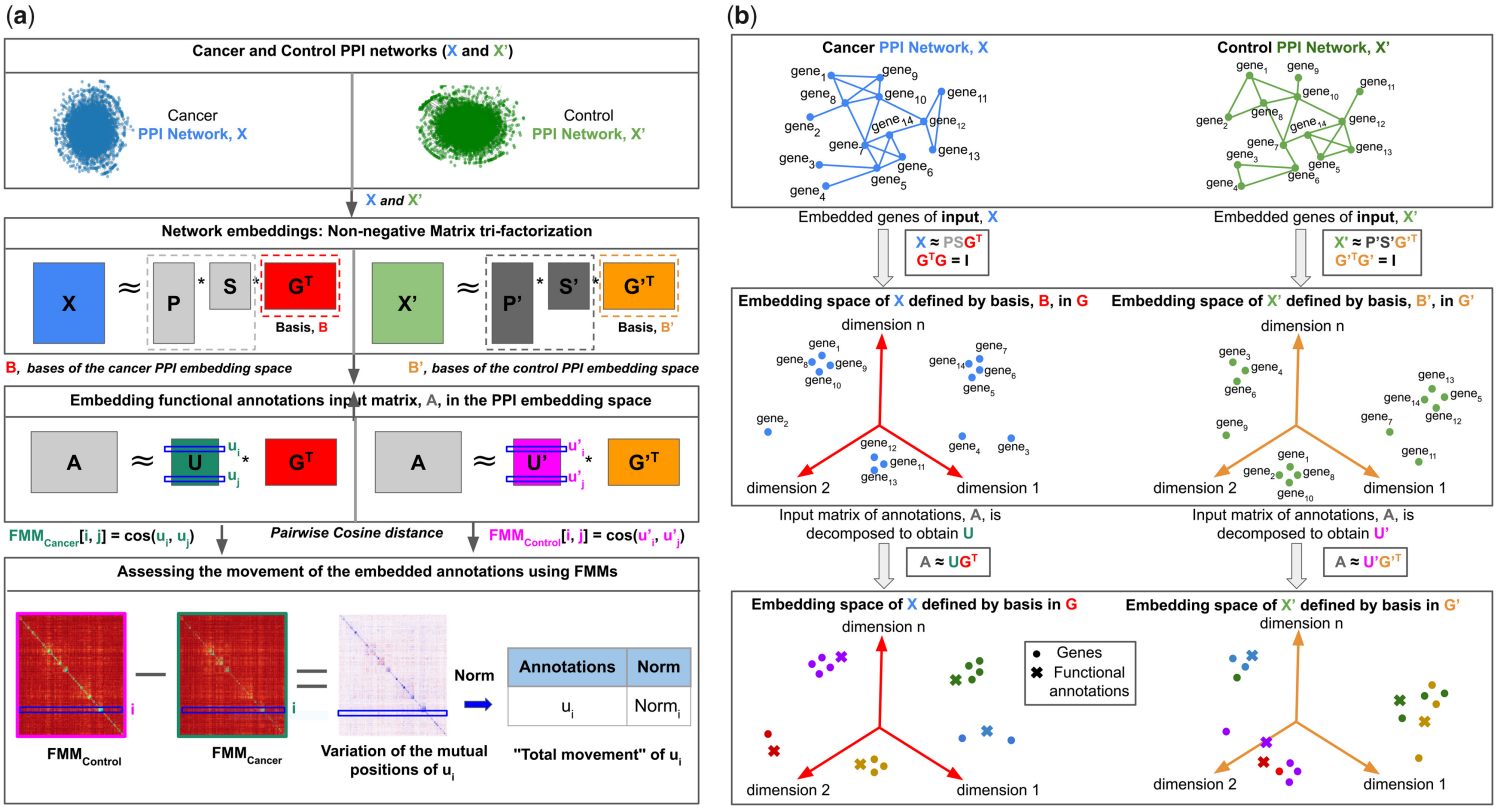
(a) Illustration of our new FMM-based method. For a pair of cancer and control tissues, we construct their tissue-specific PPI interaction networks as explained in Section 2.1 (in green and blue for cancer and control, respectively). These networks, represented by their PPMI matrices, *X* and X′, are decomposed as the products of three factors: *P*, *S*, and *G^T^* for cancer, and P′, S′, and $G\prime^{T}$ for control, where the set of all rows of *G^T^* and $G\prime^{T}$ defines the basis *B* and B′, respectively (illustrated in the second panel from the top). From these matrix factors, we use the bases matrix of the resulting NMTF-based embedding spaces, *G^T^* and $G\prime^{T}$, to generate the matrices *U* and U′, whose *i^th^* row are the embedding vectors *u_i_* of annotation *a_i_* in the cancer and control embedding spaces defined by the bases, *B* and B′, respectively (illustrated in the third panel from the top). We capture the distances (cosine distances) between the embedding vectors of all pairs of annotations, in each cancer and control embedding space, by computing FMMs as defined in Section 2.4 and illustrated at the ‘Pairwise Cosine distance’ line between the two panels at the bottom of the figure. Then, we subtract the cancer and control FMM matrices, FMM_*Control—*_FMM_*Cancer*_, to detect the changes in mutual positions of the embedding vectors *u_i_* of each annotation *a_i_* between cancer and control embedding spaces. Finally, to have the score of ‘movement’ for *u_i_* (illustrated in Section 2.7), we apply the Euclidean norm to the rows of the matrix identified as ‘Variation of the mutual positions of *u_i_*’ in the bottom panel of the figure. (b) Toy example of our new FMM-based method: the first panel shows a toy example of cancer and control PPI networks. The second panel shows a 3D illustration of the embedding spaces of the toy example of cancer and control networks generated by our NMTF framework. The third panel shows the embedding vectors of the biological functions in the aforementioned cancer and control embedding spaces. Colors in the third panel represent the biological functions of the genes.

To embed genes according to the PPMI matrix representation of a molecular network, *X*, we use NMTF to decompose *X* as the product of three non-negative factors, $X\approx P\cdot S\cdot G^{T}$, where the set of the rows of the matrix P·S defines the set of embedding vectors of the genes, *E*, and the set of the columns of *G* defines the basis, *B*, of the space in which the genes are embedded (Hu et al. 2019) (Fig. 1a illustrates the NMTF factorization on two different PPMI matrices, cancer, and control). Importantly, we apply the orthonormality constraint to the basis-defining matrix ($G^{T}G=I$), since it leads to minimal colinearities (hence, minimizing the dependencies) between the vectors of the basis, *B*, of the embedding space (Strang 2006). The decomposition is done by minimizing the function: where *F* denotes the Frobenius norm. This optimization problem is NP-hard (Ding et al. 2006); thus, we heuristically solve it by using a fixed point method that starts from an initial solution and iteratively uses multiplicative update rules (Ding et al. 2006). Such rules guarantee convergence toward a locally optimal solution that verifies the Karush-Kuhn-Tucker conditions (Ding et al. 2006) (detailed in Supplementary Section S1.1.2). To generate initial *P*, *S*, and *G* matrices, we use the Singular Value Decomposition based strategy (Qiao 2015). This strategy makes the solver deterministic and also reduces the number of iterations that are needed to achieve convergence (Qiao 2015). To measure the quality of the factorization, we compute the relative square error (RSE) between the input matrix, *X*, and its corresponding decomposition, *PSG^T^*, as $\mathrm{RSE}=\frac{||X-PSG^{T}|{|}_{F}^{2}}{X_{F}^{2}}$. We stop the iterative solver when the value of the RSE is not decreasing anymore, or after 500 iterations.


$$min_{P,S,G\geq 0}||X-PSG^{T}|{|}_{F}^{2},G^{T}G=I,$$

### 2.4 Definition of the Functional Mapping Matrix

To explore the functional organization of the gene embedding space, obtained as detailed above, we introduce the FMM. This matrix captures the mutual positions of the functional annotations that we embed in the gene embedding space. In particular, we obtain an FMM by taking as input: the matrix factor, *G*, which contains the basis, *B*, of the gene embedding space, and the relation-matrix between the genes and their functional annotations, *A*, in which entry $A[a_{i},g_{j}]$ is one if annotation *a_i_* annotates gene *g_j_*, and it is zero otherwise. First, we generate the embedding vectors of the functional annotations in the gene embedding space by decomposing the matrix *A* as the product of two matrix factors, *U* and *G^T^*, as: $A\approx UG^{T}$, where rows of matrix *U* (that we call *u_i_*) are the embedding vectors of the annotations, *a_i_*, in the gene embedding space defined by the basis, *B* (illustrated in Fig. 1 for two embedding spaces, cancer, and control). Note that, since matrix *A* is known and matrix factor *G* is computed as explained in Section 2.3, we can obtain *U* by: $U\approx {\left(G^{T}\right)}^{-1}A$, where ${\left(G^{T}\right)}^{-1}$ is the Moore-Penrose pseudoinverse of *G^T^* (Barata and Hussein 2012). Finally, the FMM is obtained by computing the pairwise cosine distances between all pairs of the embedding vectors *u_i_* of the annotations *a_i_* (the bottom panel of Fig. 1a illustrates two examples of FMMs). In particular, each entry FMM$\left[i,j]=\text{cos}(u_{i},u_{j}\right)$ corresponds to the cosine distance between the embedding vectors *u_i_* and *u_j_* of the annotations *a_i_* and *a_j_*. Thus, the resulting FMM is a symmetric distance matrix that captures the mutual positions, that henceforth we call ‘distances’, between the annotation vectors in the embedding space. We choose cosine distance over other distance measures, e.g. the dot-product, since it is a well-known normalized measure (Singhal et al. 2001), which permits direct comparison between different FMMs, i.e. we do not need any normalization step after computing the FMM.

### 2.5 Measuring the similarity of functional organization of the embedding spaces by using their Functional Mapping Matrices

For a pair of embedding spaces, we measure the similarity of their functional organization by computing the RSE between their FMMs. We use the following method to find the smallest number of dimensions, that we call the ‘optimal dimensionality’, after which the functional organization of the gene embedding spaces, as measured by the RSE between the FMMs with increasing numbers of dimensions, does not change anymore. First, we produce the gene embedding space of each cancer and control, tissue-specific PPI networks by using the NMTF algorithm (detailed in Section 2.3) with different dimensionalities (detailed in Supplementary Section S2.2.2). Then, we obtain the embedding vectors of each of the GO BP annotations in each of the cancer and the corresponding control gene embedding space and then capture the difference in the position of a GO BP annotation between cancer and control space, measured by our FMM (detailed in Section 2.4). By tracking the RSEs of the FMMs across dimensions (from 50 to 300 dimensions with a step of 50), we find that the distances of the annotation embedding vectors converge to a stable, i.e. nonchanging functional organization, after 200 dimensions for all tissue-specific PPI network embedding spaces (RSE between their FMMs plateaus, i.e. stops decreasing, see Supplementary Fig. S2). In the analysis presented below, we use the optimal dimension of the embedding space that we obtained as described here (for all tissue-specific PPI networks, their optimal dimensions are presented in Supplementary Table S5). In addition, we use this method to find the optimal dimensionality of six species-specific PPI network embedding spaces (for human, baker’s yeast, fission yeast, fruit fly, rat, and mouse), detailed in Supplementary Section S2.2.2. We apply this method to explore the similarity in the functional organization of these embedding spaces of the PPI networks of six different species (see Supplementary Section S2.2.1).

### 2.6 Evaluating the functional organization of an embedding space with its Functional Mapping Matrix

From a gene-centric perspective, an embedding space is considered to be functionally organized if genes that participate in similar biological functions are located close in the space (Gaudelet et al. 2021). This organization is commonly evaluated by applying various types of clustering methods to the embedding vectors of the genes in the space, followed by functional enrichment analyses of the genes that the clustered vectors correspond to (Malod-Dognin et al. 2019). Here, we propose to examine the functional organization of the embedding space from a function-centric perspective. Similar to the gene-centric perspective, we consider an embedding space to be functionally organized if semantically similar annotations, i.e. annotations with high Lin’s semantic similarity are embedded close in the space. To evaluate it, we apply our FMM to capture the distances of all pairs of the embedding vectors of the functional annotations in the embedding space (detailed in Section 2.4).

Then, we analyze the link between the functional similarity of the annotations, measured by their pairwiseLin’s semantic similarity, and the distances of their embedding vectors in the embedding space by performing two different experiments. We compute the Pearson’s correlation coefficient (Benesty et al. 2009) between the mutual positions of all pairs of annotation vectors in the embedding space, i.e. the cosine distances over all pairs of annotation embedding vectors, and the Lin’s semantic similarities over all pairs of annotations. Hence, a negative correlation coefficient indicates that those annotations that are embedded close in the space (lower cosine distance) tend to be functionally similar (high Lin’s semantic similarity). Also, we apply the *k*-medoid algorithm (Park and Jun 2009) to cluster the annotations based on the distances of their vectors in the embedding space, as captured by our FMM. To define the number of clusters, we use the rule of thumb (Kodinariya and Makwana 2013), $k=\sqrt{\left(n/2\right)}$, where *k* corresponds to the number of clusters and *n* to the number of annotations. Finally, we measure the intra and inter cluster Lin’s semantic similarity for the obtained clusters to assess if the annotations whose embedding vectors cluster in the embedding space are similar in biological function.

### 2.7 Quantifying the ‘movement’ of the annotation embedding vectors in cancer and control embedding spaces

We propose to quantify the changes in the mutual positions (distances), that we call ‘movement’, of the annotation embedding vectors in two different gene embedding spaces defined by bases, *B* and B′. In this study, we analyze the ‘movement’ of the annotation embedding vectors in cancer and control embedding spaces. To this end, given the pairwise cosine distances of the annotations embedding vectors in the cancer and control embedding spaces, FMM_*Cancer*_ and FMM_*Control*_, we quantify the change in the distance between two embedding vectors of annotations *u_i_* and *u_j_* as: FMM${}_{\mathrm{Control}}[i,j]$ − FMM${}_{\mathrm{Cancer}}[i,j]$. This distance is negative if *u_i_* and *u_j_* are farther in the cancer embedding space than in the control embedding space, positive if they are closer, and zero if there is no change between their positions in the embedding space of cancer and control. By taking all the pairwise distances over all *i* and *j*, FMM${}_{\mathrm{Control}}[i,j]$ − FMM${}_{\mathrm{Cancer}}[i,j]$, we define the distribution of pairwise ‘movements’ (see Supplementary Fig. S3). We define that two annotation embedding vectors, *u_i_* and *u_j_*, are ‘moving significantly apart’ in the embedding space of cancer, if their distance is greater than or equal to the $95^{th}$ percentile of the aforementioned distribution. In contrast, we define that they are ‘moving significantly closer’ in the embedding space of cancer, if their distance is smaller than or equal to the distance that corresponds to the $5^{th}$ percentile of the distribution.

To identify the annotations whose embedding vectors change the most between the cancer and control embedding spaces, first we calculate the distance between the embedding vectors of each annotation *u_i_* in the control and the cancer embedding spaces, that we call FMM${}_{\mathrm{Control}}[i]$ (which is the *i^th^* row of matrix FMM_*Control*_) and FMM${}_{\mathrm{Cancer}}[i]$ (which is the *i^th^* row of matrix FMM_*Cancer*_), respectively. So the coordinates of vector FMM${}_{\mathrm{Control}}[i]$ contains the cosine distances of *u_i_* to all other annotation embedding vectors in the control embedding space. Then, for each annotation embedding vector, *u_i_*, we define the ‘movement vector’ as D[i]=FMM${}_{\mathrm{Control}}[i]$ − FMM${}_{\mathrm{Cancer}}[i]$. Hence, the ‘movement vector’ contains the differences of the mutual positions in cancer compared with control embedding space (cosine distances) between *u_i_* and all other annotation embedding vectors. Next, we define the ‘total movement’ of annotation, *u_i_*, as the Euclidean norm of its corresponding ‘movement vector’, D[i]. In this way, for each annotation, *u_i_*, we define the score of its ‘total movement’ in cancer over control, which is high when its distance to the other annotations changes between the cancer and control embedding spaces (that we call ‘shifted’) and it is close to zero when it does not change (that we call ‘stable’). By considering the ‘total movement’ of all annotations, we define the ‘total movement distribution’ (see Supplementary Fig. S4). We consider as ‘shifted biological functions’ those functional annotations whose embedding vectors’ ‘total movement’ is two standard deviations above the mean of the ‘total movement distribution’. In contrast, we define as ‘stable biological functions’ those functional annotations whose embedding vectors’ ‘total movement’ is two standard deviations below the mean of the ‘total movement’ distribution.

### 2.8 Distances between the embedded entities in the embedding space

We use the cosine distance to determine the distance between the embedding vectors of two entities (genes or functions in this study) in the same gene embedding space defined by basis *B*. We recall that in the embedding space defined by *B*, the embedding vector of gene *g_i_* is the *i^th^* row of matrix P·S, and that the embedding vector of annotation *a_j_* is the *j^th^* row of matrix *U* (detailed in Section 2.4 and illustrated in Fig. 1b). Before using the cosine distance, we confirm that the embedding vectors of the biological functions (GO BP terms) are significantly closer in space to the embedding vectors in the same space of the genes that they annotate than to the embedding vectors of other genes (Mann–Whitney *U P*-value ≤.05, see Supplementary Table S12). This confirms that annotations and genes are functionally organized in the embedding space.

## 3 Results and discussion

Inspired by Malod-Dognin et al. (2019) who, in a gene-centric analysis, observed that cancer-related genes are the most rewired between cancer and control embedding spaces and used this property to predict novel cancer-related genes, we use our FMM-based method to confirm that the embedding spaces of both, cancer and control, are functionally organized and that this organization changes between cancer and control (Section 3.1). Then, we find that the embedding vectors of well-known cancer-related functions move the most between cancer and control compared with embedding vectors of other annotations (Section 3.1). We exploit this observation to predict new cancer-related functions, which we validate by analysis of their enrichment in known cancer-related functions (detailed below), automatic literature search, and manual literature curation for the most promising predictions (Section 3.2). Moreover, we go beyond and exploit the ‘movement’ of the annotation embedding vectors to predict new cancer-related genes (Section 3.3), finding four new cancer-related genes, which we validate by literature curation and retrospective analyses of patient survival, but whose role with cancer has yet to be experimentally validated.

### 3.1 Cancer alters the functional organization of the healthy cell embedding space

Here, we focus on applying our FMM-based method to confirm that the embedding spaces of both, cancer and control, are functionally organized (detailed in Section 2.6). To this end, we generate the embedding spaces of the most prevalent cancers (breast, prostate, lung, and colorectal cancer) and their control tissues (breast glandular cells, prostate glandular cells, lung pneumocytes, and colorectal glandular cells) by applying the NMTF algorithm on the corresponding tissue-specific PPI networks (detailed in Sections 2.1 and 2.3). Then, we use our FMM-based method to embed GO BP terms into these gene embedding spaces and to capture their distances over the cancer and control embedding spaces (detailed in Section 2.4). By analyzing the FMM of each embedding space, we find that the annotation embedding vectors that cluster together based on their cosine distances in each space have, on average, Lin’s semantic similarity 1.32 times larger than those that do not cluster together in space (see column ‘Fold’ in Table 1 for the corresponding results for each embedding). Note that our FMM-based cluster analysis is needed, since none of the standardly used techniques, including t-SNE, UMAP, and MDS, gives good clustering results (see Supplementary Figs. S5–S7). Hence, the GO BP terms corresponding to the embedding vectors that cluster together in space are more functionally related than those whose embedding vectors do not cluster in space (see Table 1). Thus, both cancer and control embedding spaces are functionally organized. We further confirm this conclusion by comparing these results against a randomized experiment, i.e. when randomly rewiring the PPI networks (detailed in Supplementary Section S1.2.4). As expected, we find that annotations whose embedding vectors are close in these randomized spaces are not more functionally similar (as measured by the Lin’s semantic similarity) than those whose embedding vectors are far in the space, i.e. they are not functionally organized in the randomized space (see Table 1 and Supplementary Table S6).

**Table 1.** The embedding spaces of the most prevalent cancers (breast, prostate, lung, and colorectal cancer) and their control tissues (breast glandular cells, prostate glandular cells, lung pneumocytes, and colorectal glandular cells) are functionally organized.

Embedding	Intra	Inter	Fold	*P*-value
Control breast	0.22	0.17	1.29	$2.12\times 10^{-6}$
Cancer breast	0.23	0.16	1.43	$2.68\times 10^{-5}$
Control prostate	0.24	0.17	1.41	$2.24\times 10^{-6}$
Cancer prostate	0.21	0.15	1.40	$1.04\times 10^{-6}$
Control colon	0.19	0.16	1.18	$4.04\times 10^{-3}$
Cancer colon	0.21	0.16	1.31	$1.68\times 10^{-5}$
Control lung	0.19	0.17	1.11	$2.17\times 10^{-4}$
Cancer lung	0.22	0.15	1.46	$5.32\times 10^{-6}$
Random example	0.17	0.17	1.00	0.14

The first column, ‘Embedding’, lists the tissues. The second column, ‘Intra’, shows the average Lin’s semantic similarity of those annotations whose embedding vectors cluster together based on their cosine distances in the embedding space. The third column, ‘Inter’, shows the average Lin’s semantic similarity of those annotations whose embedding vectors do not cluster together based on their cosine distances in the embedding space. The fourth column, ‘Fold’, displays how many times the average Lin’s semantic similarity of those annotations whose embedding vectors cluster together based on their cosine distances in the embedding space is higher than of those annotations whose embedding vectors do not cluster together. The fifth column, ‘*P*-value’, shows the *P*-value from a one-sided Mann–Whitney *U* test comparing the Lin’s semantic similarity between annotations whose embedding vectors cluster together and those with non-clustered embedding vectors. This table also includes an example of a randomly rewired PPI network (Random Example). The complete information with all the random tissue-specific PPI networks can be found in Supplementary Table 6.

Having confirmed that both embedding spaces, cancer, and control, for all four cancers, are functionally organized, we investigate if this organization changes between them. To do so, we assess if there are pairs of annotation embedding vectors whose distances in the embedding space are significantly altered in cancers (detailed in Section 2.7). For the four studied cancers, we find an average of 72,326 (5% of the total number) of pairs that move significantly closer in the cancer space compared with control (see Supplementary Fig. S8 for an illustration of this variation). We find that this set of pairs (that are closer) are 1.3 times closer in the cancer space than in the control one. Similarly, we find the same percentage of pairs that move significantly apart in the cancer embedding space compared with control. Here, we find that this set of pairs (that move apart) are 1.4 times farther in the cancer space in comparison to the control one. In conclusion, these results demonstrate that cancer alters the functional organization of the healthy (control) cell.

We have shown above that cancer alters the functional organization of the control PPI network embedding space by changing the distances of the annotation embedding vectors in the space. Now, we investigate how this change is related to cancer (and if it can be used to predict novel cancer-related functions). We use our FMM-based methodology to identify the annotation embedding vectors that change their distances (that we call ‘movement’) between cancer and control embedding spaces. Then, we compare the ‘movement’ of our set of cancer-related functions and the rest of the annotations. Interestingly, we observe that the embedding vectors of cancer-related functions move the most between cancer and control embedding spaces compared with those of other annotations. Indeed, these annotation vectors move on average 2.4 times more than the rest of the annotation embedding vectors in all four cancers (Mann–Whitney *U* test with *P*-value <.05). This suggests that the ‘movement’ of the annotation vectors is related to cancer, i.e. it could be exploited to find new cancer-related functions (presented in the next section).

### 3.2 The ‘movement’ of the annotations in the embedding space predicts cancer-related functions

**Figure 2.**
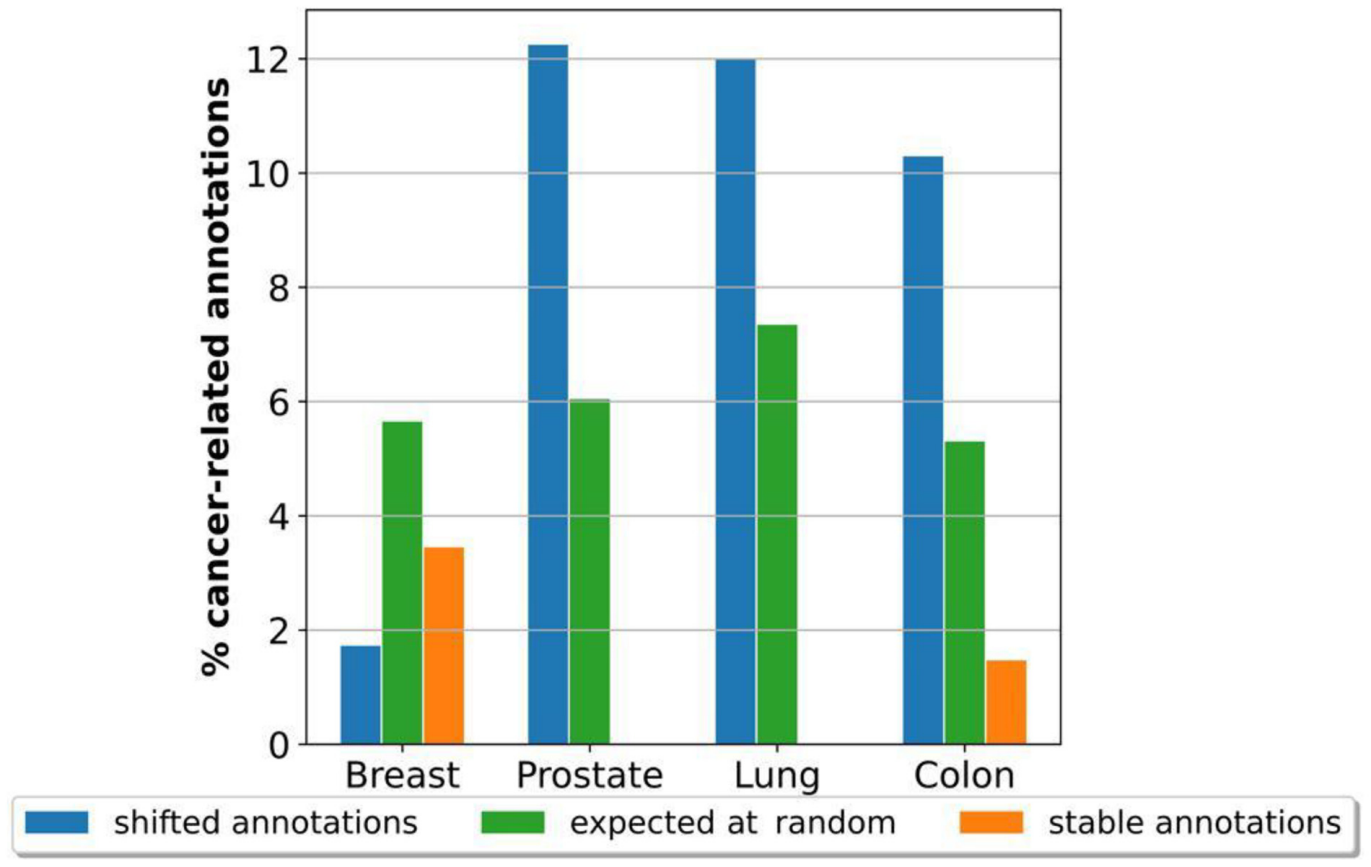
‘Movement’ in the embedding space is related to cancer. The panel contains the percentages of enriched cancer-related GO BP terms out of all GO BP terms (vertical axis) in the ‘shifted’ annotations set (in blue), ‘stable’ annotations set (in orange), and the expected by random (in green), for each cancer type (on the horizontal axis).

Here, we exploit the ‘movement’ of the annotations’ vectors to predict novel cancer-related functions. Following the approach detailed in Section 2.7, we find two groups of annotations based on their ‘movement’: ‘shifted’ and ‘stable’ group of annotations (the numbers of GO BP annotations in the two sets for each of the four cancers are presented in Supplementary Table S7). For these sets of annotations, we perform the hypergeometric test (with *alpha *= .05, Rice 2006) to assess if they have significantly more, or significantly less cancer-related functions than the background set of genes (the background set of genes contains all genes that are in the corresponding tissue-specific PPI network). We observe that for three out of four cancers, the ‘shifted’ annotations are significantly enriched in cancer-related functions (*P*-value of .85, .02, .02, and .04 for breast, colorectal, prostate, and lung, respectively). In contrast, the ‘stable’ annotations are significantly depleted in these functions (*P*-value of .49, .88, .80, and .68, for breast, colorectal, prostate, and lung, respectively), i.e. they have a significantly lower percentage of cancer-related functions than the background (see Fig. 2). This observation does not hold only for the ‘shifted’ annotations of breast cancer (*P*-value of .85). This discrepancy can be attributed to the type of cancer samples used in this analysis and to our definition of cancer-related annotations. While the TCGA’s samples of colorectal, lung, and prostate are mostly from adenocarcinomas, over 99% of the TCGA’s samples of breast cancer are from neoplasms (see Supplementary Table S1). Indeed, as detailed in Section 2.2, we use the COSMIC oncogenes to define our cancer-related GO BP terms. These oncogenes are mainly defined from adenocarcinomas samples; in particular, for breast cancer, only 8% of the samples in COSMIC come from neoplasms, while in TCGA, over 99% of the samples come from neoplasms. This highlights the importance of improving the definition of cancer-related functions to include different types of cancer of the same organ.

Although the shifted set of annotations is significantly enriched in our cancer-related annotations, we notice that most of the annotations in this set are not cancer-related. In particular, we find that only 2 (2%), 5 (12%), 5 (10%), and 6 (10%) of the annotations in the ‘shifted’ set are cancer-related for breast, prostate, lung, and colorectal cancer, respectively (see Supplementary Tables S8–11). Thus, to validate the remaining unknown to be cancer-related annotations, we extend the systematic approach used in the study by Ceddia et al. (2020) and conduct a systematic literature search in the PubMed database (Geer et al. 2010). We automatically retrieve the number of scientific publications that associate each GO BP term with a specific cancer type. To do so, we search for co-occurrences between the GO BP term and the cancer type in the abstracts of PubMed publications. We find that 33 (58%), 31 (65%), 29 (63%), and 36 (52%) of these annotations have at least one publication demonstrating their role in breast, lung, prostate, and colorectal cancer, respectively. These high percentages of literature validation indicate that the remaining annotations, which we could not validate in the currently available literature, are candidates for novel cancer-related functions.

Finally, we do manual literature curation for the most promising predictions identified above. In particular, we rank the predictions by the magnitude of their ‘movement’ and we investigate the top 10 most ‘moved’ ones. We detect that, although these functions are not reported in the literature to be directly related to cancer, their link with cancer is clear: for instance, we find ‘the positive regulation of activated T cell proliferation’ in breast cancer (see Supplementary Table S8). This is a well-known regulation process in breast cancer development, and it could be connected to the ‘cooperation’ of breast cancer cells with the immune cells (Chang and Beatty 2020). Other examples include ‘cleavage furrow formation’ and ‘mitotic spindle midzone assembly’ in prostate and colorectal cancers, respectively (see Supplementary Tables S9 and 11). The failure of these processes during cell division has been associated with carcinogenesis (Sagona and Stenmark 2010; Ganem et al. 2007). Finally, we find ‘the positive regulation of endodeoxyribonuclease activity’ in lung cancer. A deficiency in this process is linked with most of the mutations and genomic alterations that are relevant to cancer (Hoeijmakers 2009). An extended discussion for the rest of the annotations in each cancer type top 10 predictions can be found in Supplementary Section S1.2.5. Finally, we also find eight common functions that are ‘shifted’ in all four cancers (detailed in Supplementary Section S1.2.6). We observe that these functions describe general mechanisms of cancer, e.g. activation of the stress-activated MAPK cascade, and are closely related to the cancer hallmarks (Hanahan and Weinberg 2011). This suggests that our analysis could be extended to more cancers to uncover new pan-cancer functions.

### 3.3 The ‘movement’ of cancer-related annotations in the embedding spaces predicts oncogenic genes

In this section, we investigate if the functions that are shifted in cancer (compared with control) can be used to identify novel cancer-related genes. To this aim, we first demonstrate that the embedding space captures the functions of a given gene by placing its embedding vector close (low cosine distance) to the embedding vectors of those GO BP terms that describe the gene’s biological functions (detailed in Section 2.8). We hypothesize that the alteration in the cosine distance between the gene embedding vector and the GO BP embedding vector may indicate that the gene is losing a function (if the distance increases), or that the gene is gaining a function (if the distance decreases). Hence, we prioritize as cancer-related those genes whose embedding vectors change their distances to the vectorial representations of the ‘shifted’ functions in the embedding space the most.

To evaluate this hypothesis, we first assess if ‘literature-validated’ genes (see the definition below) change significantly more their distances to our ‘shifted’ functions than the background genes in the cancer space compared with control. To this end, similar to the methods explained in Section 2.7, for each gene, we compute a vector with *n* positions, where *n* corresponds to the number of the ‘shifted’ GO terms and in which each entry corresponds to the ‘movement’ (change in mutual positions) of the gene and the GO term. Since this ‘movement’ is bi-directional (getting closer or further), we use the absolute value of the ‘movement’ at each coordinate of this vector, to keep only the magnitude of this ‘movement’ independently of the direction of the ‘movement’. Then, since all the values in the *n*-dimensional vector are now positive, for each gene we assign as its cancer-related score the maximum value (maximum magnitude of movement) in its corresponding vector. Hence, we define the maximum ‘movement distribution’ of the gene embedding vectors as the set of all aforementioned maximum values of ‘movement’. For each cancer type, we consider as ‘literature-validated’ the genes with at least one publication in PubMed indicating their role in the corresponding cancer type. To do this evaluation, we apply the same systematic approach as the one used to validate the ‘shifted’ annotations in Section 3.2. In all four cancers, we find that ‘literature-validated’ genes ‘move’ significantly more toward or away (higher cancer-related score) from our ‘shifted’ functions than the background genes (we compare these two ‘movement’ distributions with Mann–Whitney *U* test with *P*-value <.05). Thus, we use this property to predict new cancer-related genes. We predict as cancer-related those genes that are above or at the 95th percentile of the maximum ‘movement’ distribution (see Supplementary Fig. S9). In this way, we predict as cancer-related 346, 234, 325, and 379 genes in breast, lung, prostate, and colorectal cancer, respectively, which we call ‘shifted genes’. In the rest of this section, we validate these predicted cancer-related genes in two ways: systematic literature curation and by retrospective analyses of patient survival curves (detailed below).

We validate in the literature that 233 out of 346 (67%), 144 out of 234 (61%), 179 out of 325 (55%), and 187 out of 379 (49%) of our predictions are cancer-related in breast, lung, prostate, and colorectal cancer, respectively. Indeed, among our literature-validated predictions, we find well-known cancer genes, i.e. BRAF in breast cancer (225 publications), CASP8 in lung cancer (123 publications), or MSH6 in colorectal cancer (205 publications). Also, we assess if our cancer gene predictions are prognostic markers of patient survival, which we measure with patient survival curves (we collected the data from the HPA, Pontén et al. 2008). We find that 16 (4.6%), 7 (2.9%), 4 (1.2%), and 17 (4.4%) of these genes are registered in the HPA as breast, lung, prostate, and colorectal cancer prognostic markers, respectively. Since these survival curves are based on differential gene expression analyses (Kim et al. 2020), we hypothesize that our method prioritizes genes that are not differentially expressed. Indeed, only 38 (11%), 85 (36%), 19 (6%), and 56 (15%) of our predicted cancer-related genes are deferentially expressed in breast, lung, prostate, and colorectal cancer tissues with respect to their corresponding control tissues, respectively (using expression data from TCGA projects, as detailed in Supplementary Table S1). These results align with Malod-Dognin et al. (2019), who demonstrated that there exist important cancer-related genes (validated by wet-lab experiments) that are not differentially expressed in control and cancer. We hypothesize that the role of these genes in cancer could be connected with post-translational modifications of their expressed proteins. These modifications modulate the functions and interactions of the proteins after translation (Thygesen et al. 2018) and have been reported in several cancer types, e.g. ovarian cancer (Shetty et al. 2012) or skin cancer (Povlsen et al. 2012). In conclusion, our method identifies genes whose transcriptional patterns have not changed and thus is complementary to the traditional differential expression analysis.

Finally, we go beyond the above validation and focus on the top 10 ‘shifted’ genes (the most shifted ones) of each cancer type. We largely validate these top 10 shifted genes, either as cancer biomarkers (of prognosis) or as cancer-related in the literature (see Table 2 and Supplementary Tables S13–15). Thus, we conjecture that the remaining four nonvalidated genes (PRDM11 in lung cancer, C9orf72 and MINDY3 in prostate cancer, and H4C6 in colorectal cancer) are also cancer-related. Indeed, PRDM11 is part of a broad family of transcriptional regulators, several of which are deregulated in cancer (Fog et al. 2015). It is highly expressed in the lungs, as well as in peripheral blood immune system cells. Although it has been linked with the enhancement of lymphomagenesis (Fog et al. 2015), our study is the first one to suggest its role in lung cancer. Another example is MINDY3 in prostate cancer; MINDY3 codes for a protein that contains a caspase-associated recruitment domain and may be involved in apoptosis (Safran et al. 2010). Even though it has been identified as a tumor suppressor in lung and gastric cancers (Lu et al. 2014), our study is the first to link it with prostate cancer. For the same cancer type, prostate cancer, we find C9orf72, a gene that has been associated with several neurodegenerative disorders (McCauley et al. 2020). Although its role in cancer is unknown, its participation in important cancer-related processes, such as autophagy (Fog et al. 2015) and inflammation (The UniProt Consortium 2015), support our observation that it may be cancer-related. Finally, we predict gene H4C6 as being involved in colorectal cancer, which is a member of the histone H4 family that encodes a replication-dependent histone. Although no publication relates this gene to cancer, its involvement in cellular senescence and mitotic prophase (Safran et al. 2010) suggests that this gene may have an important role in cancer progression. In conclusion, we introduce a method to predict new cancer-related genes based on their distance to the most ‘shifted’ functional annotations in cancer over control molecular network embedding space. We validate our predictions of new cancer-related genes through literature curation and retrospective analyses of patient survival data. Importantly, these new predicted cancer-related genes cannot be identified by using the traditional differential-expression analysis.

**Table 2.** Top 10 shifted genes (the most shifted ones) in prostate cancer.

Gene name	PubMed counts	Pan-cancer prognostic marker
C9orf72	0	0
PIK3R2	2	0
TAF13	0	2
MINDY3	0	0
EIF5B	1	3
SSB	7	3
SGSM3	0	1
NKX3-1	314	0
RPS4X	0	2
FAM204A	0	1

The first column, ‘Gene name’, presents the gene names of the top 10 ‘shifted’ genes. The second column, ‘PubMed Counts’, contains the number of publications in PubMed that relate the gene to prostate cancer. The third column, ‘Pan-Cancer Prognostic Marker’, indicates for how many cancer types the gene is considered to be a prognostic marker based on survival curves collected from the Human Protein Atlas (Pontén *et al.*, 2008).

## 4 Conclusion

By introducing our new FMM methodology, we initiate the investigation of the embedding spaces of the tissue- and disease-specific molecular networks from a functional point of view. In Supplementary Section S1.2.2, we demonstrate that our FMM methodology better captures the functional interaction between GO BP terms than the traditional gene-centric approach. We show that our FMM can efficiently be applied to address different problems, i.e. to find the optimal dimensionality of the embedding space, to analyze the similarities between the functional organization of different embedding spaces (in this study, those corresponding to cancer and control), or to find the functional changes produced by cancer. Moreover, we use our method to predict four new cancer-related genes for which we found some literature indicating their involvement in cancer, but whose role in cancer has yet to be experimentally validated. Furthermore, our methodology could be easily applied to other bioinformatics tasks, such as patient and tissue stratification, or to uncover evolutionary similarities. In the context of evolutionary similarities, we apply our FMM methodology to capture the functional organization of six species-specific PPI network embedding spaces and find that it correctly identifies the evolutionary connections between the species (detailed in Supplementary Section S2.2.1). Moreover, in Supplementary Section S1.2.3, we demonstrate that our FMM captures the hierarchical organizations of the GO BP terms in network embedding spaces. However, extracting novel knowledge from that higher-level organization is left for future study. Finally, our new methodology is generic and can be applied to any discipline that analyzes embedded network data in which the embedded network nodes can be functionally annotated, e.g. social, or economic networks, paving the road to new algorithms for mining the data by utilizing the embedding space from a functional perspective.

## Supplementary Material

btad281_Supplementary_Data
