# Supplementary material for: A functional analysis of omic network embedding spaces reveals key altered functions in cancer
Source: Bioinformatics. 2023 Apr 21;39(5):btad281. doi: 10.1093/bioinformatics/btad281 (PMC10172038; doi:10.1093/bioinformatics/btad281)
Supplement: btad281_Supplementary_Data [file btad281_supplementary_data.pdf]

Supplementary Material for A functional analysis of omic network embedding spaces reveals key altered functions in cancer

Sergio Doria-Belenguer[1], Alexandros Xenos[1], Gaia Ceddia[1], Noël Malod-Dognin[1,2] and Nataša Pržulj[1,2,3]
[1]Barcelona Supercomputing Center (BSC), 08034 Barcelona, Spain.
[2]Department of Computer Science, University College London, WC1E 6BT London, United Kingdom.
[3]ICREA, Pg. Lluís Companys 23, 08010 Barcelona, Spain.

This Supplementary Material is divided in two sections: "Cancer-related application," and "Species-related application." The first section starts with a subsection titled "Supplementary Materials," which has subsections "Summarizing functional annotations into functional clusters," and "Multiplicative update rules." It continues with subsections titled "Supplementary Results," which contains subsections "Impact of the PPI network matrix representation to the functional organization of the embedding space," "Our FMM-based methodology captures more biological information from the embedding space compared to the actual gene-centric approaches," "The FMMs reveal the higher-order functional organizations of the GO BP terms in the network embedding spaces," "FMM discriminates between functionally and not functionally organized embedding spaces," "FMMs identify novel cancer-related functions," and "Towards pan-cancer functions." Finally, the first section ends with subsections containing Supplementary Figures and Supplementary Tables. The second section starts with a subsection titled "Supplementary Materials," which has subsection "Species-specific PPI networks." It continues with subsections titled "Supplementary Results," which contains subsections "FMM captures the functional organization of different species-specific embedding spaces," and "The similarity between the FMMs of different dimensional spaces reveal the optimal dimensionality of the embedding space." Finally, the second section ends with subsections containing Supplementary Figures and Supplementary Tables.

Contents

1 Cancer-related application **4**

1.1 Supplementary Materials and Methods 4

1.1.1 Summarizing functional annotations into functional clusters 4

1.1.2 Multiplicative update rules 4

1.2 Supplementary Results 6

1.2.1 Impact of the PPI network matrix representation to the functional organization of the embedding space 6

1.2.2 Our FMM-based methodology captures more biological information from the embedding space compared to the actual gene-centric approaches 8

1.2.3 The FMMs reveal the higher-order functional organizations of the GO BP terms in the network embedding spaces 10

1.2.4 FMM discriminates between functionally and not functionally organized embedding spaces 11

1.2.5 FMMs identify novel cancer-related functions 12

1.2.6 Towards pan-cancer functions 13

1.3 Supplementary Figures 16

1.4 Supplementary Tables 31

2 Species-related application **42**

2.1 Supplementary Materials and Methods 42

2.1.1 Species-specific PPI networks 42

2.2 Supplementary Results 44

2.2.1 FMM captures the functional organization of different species-specific embedding spaces 44

2.2.2 The similarity between the FMMs of different dimensional spaces reveal the optimal dimensionality of the embedding space 46

2.3 Supplementary Figures 48

2.4 Supplementary Tables 52

1 Cancer-related application

1.1 Supplementary Materials and Methods

1.1.1 Summarizing functional annotations into functional clusters

The analysis of a large set of functional annotations is not straightforward. These large sets typically contain redundant and dependent annotations that can be summarized to improve the biological interpretation of the sets. To summarize them, different tools, e.g., REVIGO (Supek *et al.*, 2011) or DAVID (Dennis *et al.*, 2003), have been proposed. These methods group similar functional annotations by computing their Lin's semantic similarity. Thus, large groups of annotations are summarized into functional clusters according to different semantic similarity metrics, e.g., the simRel score (Schlicker *et al.*, 2006) or the Lin's semantic similarity (Lin *et al.*, 1998). In this paper, we propose to use the REVIGO tool to summarize a large list of annotations. As a first step, we use REVIGO (with the default parameters) to cluster the functional annotations based on their Lin's semantic similarity. Then, we consider as the most representative function of each cluster, the annotation terms having the highest average Lin's semantic similarity with the other terms in the cluster. In this manuscript, we use this method to summarize the functions altered by cancer.

1.1.2 Multiplicative update rules

As presented in section 2.3 of the main paper, the Non-negative Matrix Tri-Factorization, NMTF, can be formulated as the following minimization problem:

$$\min_{P,S,G\geq 0} f(P,S,G) = min_{P,S,G\geq 0}\|X - PSG^T\|_F^2, G^TG = I,$$

where F denotes the Frobenius norm, X is the PPMI matrix representation of a molecular network (whose nodes are genes), rows in matrix $P \cdot S$ are the embedding vectors of the genes and columns in G^T are the axis of the basis describing the space in which the genes are embedded.

Following the semi-NMTF simplification Ding *et al.* (2008) for a more computationally tractable

solution, we remove the non-negativity constraint on $S \geq 0$. To solve the optimization problem, we derive the Karush-Kuhn-Tucker (KKT) conditions for our NMTF as follows:

$$
\begin{aligned}
\frac{\partial f}{\partial G} = -X^T PS + GS^T P^T PS - \eta_1 = 0, \\
\frac{\partial f}{\partial S} = -P^T XG + P^T PSG^T G = 0, \\
\frac{\partial f}{\partial P} = -XGS^T + PSG^T GS^T - \eta_2, \\
\eta_1, G \geq 0, \\
\eta_1 \odot G = 0, \\
\eta_2, P \geq 0, \\
\eta_2 \odot P = 0,
\end{aligned}
$$

where $\odot$ is the Hadamard (element wise) product and matrices η_1 and η_2 are the dual variables for the primal constraint $G, P \geq 0$. For S, we have the following closed formula:

$$
S = (P^T P)^{-1}(P^T MG)(G^T G)^{-1} \tag{1}
$$

As explained in (Pržulj, 2019), we derive the following multiplicative update rule to solve the KKT conditions above:

$$
\begin{aligned}
G_{ij} \leftarrow G_{ij}\sqrt{\frac{(X^T PS)^+_{ij} + G(S^T P^T PS)^-_{ij}}{(X^T PS)^-_{ij} + G(S^T P^T PS)^+_{ij}}} \\
P_{ij} \leftarrow P_{ij}\sqrt{\frac{(XGS^T)^+_{ij} + P(SG^T GS^T)^-_{ij}}{(XGS^T)^-_{ij} + P(SG^T GS^T)^+_{ij}}}.
\end{aligned} \tag{2}
$$

We start from initial solutions, G_{init}, P_{init}, S_{init}, and iteratively use Equations (1) and (2) to compute new matrix factors G, P and S until convergence. To generate initial G_{init}, P_{init} and S_{init}, we use the Singular Value Decomposition based strategy (Qiao, 2015). However, SVD matrix factors can contain negative entries; thus, we use only their positive entries and replace the negative entries with 0, to account for the non-negativity constraint of the NMTF. This strategy makes

the solver deterministic and also reduces the number of iterations that are needed to achieve convergence (Qiao, 2015).

We measure the quality of the factorization by sum of the relative square errors (RSE) between the decomposed matrices and the corresponding decompositions:

$$RSE = \frac{||X - PSG^T||_F^2}{||X||_F^2}.$$

In our implementation, the iterative solver stops after 1000 iterations, the value for which the RSE of the decomposition is not decreasing anymore.

1.2 Supplementary Results

1.2.1 Impact of the PPI network matrix representation to the functional organization of the embedding space

In this section, we compare the ability of the adjacency and PPMI matrix representations of the tissues-specific PPI networks (detailed in sections 2.1 of the main text) to produce functionally coherent network embedding spaces. To this aim, we embed each tissue-specific PPI network by applying our NMTF-based methodology (see section 2.3 of the main text) on either its adjacency matrix representation or on its PPMI matrix representation. We generate these embedding spaces with 200 dimensions since this dimensionality corresponds to the optimal dimensionality of such spaces (as detailed in section 2.5 of the main text).

In a first step, as standardly done in the literature, we compare the ability of the adjacency and PPMI matrix representations to produce functionally coherent embedding spaces from the gene-centric point of view. For each embedding space, we cluster together genes that are embedded close in space by applying the k-medoid algorithm (Park and Jun, 2009) on the genes' embedding vectors. For the number of clusters, we use the heuristic rule of thumb ($k = \sqrt{\frac{n}{2}}$, where n is the number of nodes in the tissue-specific network) (Kodinariya and Makwana, 2013). We end up with 65, 45, 44, 44, 42, 38, 47, and 47 clusters for breast cancer, breast glandular cells, prostate cancer, prostate glandular cells, lung cancer, lung pneumocytes, colorectal cancer, and colorectal glandular cells,

respectively. After clustering, we measure the enrichment of those clusters in GO BP annotations by using the sampling without replacement strategy (hypergeometric test) and we consider a GO BP term to be significantly enriched in a gene cluster if the corresponding enrichment p-value, after Benjamini Hochberg correction for multiple hypothesis testing (Benjamini and Hochberg, 1995), is smaller than or equal to 5%. For each embedding space, we report the percentage of enriched clusters (clusters with at least one enriched GO BP term), the percentage of enriched genes (genes that are annotated with at least one GO BP term that is enriched in their clusters), and the percentage of enriched GO BP terms. As detailed in Supplementary Table 3, we find that the embedding spaces obtained from the PPMI matrix representations are functionally more coherent, with 74.80% of enriched clusters, 22.87% of enriched genes and 51.10% of enriched GO BP terms (on average over the eight tissues-specific PPI networks), compared to the embedding spaces that are obtained from the adjacency matrix representations (with 71.33% of enriched clusters, 16.23% of enriched genes and 37.56% of enriched GO BP terms on average).

In a second step, we compare the ability of the adjacency and PPMI matrix representations to produce functionally coherent network embedding spaces from our new function-centric point of view. To this aim, we use our FMM-based method to embed and capture the relative positions of the GO BP terms in the eight tissues-specific PPI network embedding spaces described above (detailed in section 2.4 of the main text). We evaluate the functional organization of these embedding spaces by assessing if functionally similar GO BP terms (with high Lin's semantic similarity) are located close in the embedding space, and thus have low values in the corresponding FMM. To this aim, we first compute the pairwise Lin's semantic similarity (Lin *et al.*, 1998) between any two GO BP terms. Then, we cluster GO BP terms based on their proximity in the embedding space (detailed in section 2.6 of the main text) and report both the average semantic similarity of the pairs of GO BP terms that are in the same cluster ("intra-SS") and the average semantic similarity of the pairs of GO BP terms that are not clustered together ("inter-SS"). Intuitively, the higher the intra-SS and the lower the inter-SS, the better functionally organized the embedding space is. As detailed in Table 1 and Supplementary Table 4, we find that the embedding spaces obtained from the PPMI matrix representations are more functionally coherent, with an intra-SS of 0.21 and an inter-SS of

0.161 (on average over the eight tissues-specific PPI networks), compared to the embedding spaces obtained from the adjacency matrix representations (with an intra-SS of 0.18 and an inter-SS of 0.165, on average).

Furthermore, for each tissues-specific PPI network, the pairs of GO BP terms that are clustered together in the PPMI-based network embedding spaces have statistically significantly higher Lin's semantic similarity than the pairs of GO BP terms that are clustered together in the adjacency-based network embedding spaces (with all one-sided Mann-Whitney U test p-values being smaller than or equal to 4×10^{-3}, as detailed in Supplementary Table 4).

To conclude, both the gene-centric and our FMM approach show that the embedding spaces obtained from the PPMI matrix representations of our tissues-specific PPI networks better capture the cell's functional organization than the embedding spaces obtained from the adjacency matrix representations of these networks. These results further demonstrate that the PPMI matrix is not only a richer representation compared to the adjacency matrix (Xenos *et al.*, 2021), but also that the extra information that it contains is useful for producing a more functionally organized embedding space.

1.2.2 Our FMM-based methodology captures more biological information from the embedding space compared to the actual gene-centric approaches

In this section, we compare the ability of our FMM-based method to uncover functional interactions between GO BP terms from the PPI network embedding spaces to that of the standard gene-centric approach. To this aim, we consider the eight cancer and control tissues-specific PPI networks described in section 2.1 of the main text, which we embed by applying our NMTF-based methodology on their PPMI matrix representations (see section 2.3 of the main text). We generate these embedding spaces with 200 dimensions since this dimensionality corresponds to the optimal dimensionality of such spaces (as detailed in section 2.5 of the main text).

For a given tissues-specific PPI network embedding space, our FMM directly quantifies all the functional interactions between any two GO BP terms that annotate genes in the PPI network by measuring the cosine distance between the GO BP terms' embedding vectors (see section 2.4 of the

main text). On the other hand, the gene-centric approach does not directly uncover such functional interactions between GO BP terms. Instead, we indirectly uncover them by performing the following gene clustering and enrichment analysis. For each embedding space, we cluster together genes that are embedded close in space by applying the k-medoid algorithm (Park and Jun, 2009) on the genes' embedding vectors. For the number of clusters, we use the heuristic rule of thumb ($k = \sqrt{\frac{n}{2}}$, where n is the number of nodes in the tissue-specific network) (Kodinariya and Makwana, 2013). We end up with 65, 45, 44, 44, 42, 38, 47, and 47 clusters for breast cancer, breast glandular cells, prostate cancer, prostate glandular cells, lung cancer, lung pneumocytes, colorectal cancer and colorectal glandular cells, respectively. Then, we measure the enrichment of the resulting gene clusters in GO BP terms by using the sampling without replacement strategy (hypergeometric test) and we consider a GO BP term to be significantly enriched in a gene cluster if the corresponding enrichment p-value, after Benjamini and Hochberg correction for multiple hypothesis testing (Benjamini and Hochberg, 1995), is smaller than or equal to 5%. Then, we consider that two GO BP terms functionally interact if they are both significantly enriched in the same gene cluster. Finally, for the GO BP terms that are significantly enriched in at least one gene cluster, we measure the agreement between the functional interactions uncovered by the gene-centric approach and the functional interactions that are captured by our FMM methodology by using the following receiver operating characteristic (ROC) curve analysis. In particular, for each GO BP pair, we consider the result of the gene-centric approach as the ground truth, i.e., a pair of GO BP terms is considered as "true" if the two terms are enriched in the same cluster, or as "false" otherwise. Also, for each GO BP pair, we consider as the prediction score their cosine similarity in the embedding space (1 minus their associated value in the FMM). Then, we compute the area under the ROC curve (AUROC) (Bradley, 1997) between the ground truth and the prediction score over all the considered GO BP pairs. Note that an AUROC score of 0.5 corresponds to a random classification and a score of 1 to a perfect one. Hence, the closer to one the AUROC score is, the higher the agreement between our FMM-based method and the gene-centric approach.

On average over our eight tissues-specific PPI networks, we find that only 51.1% of the GO BP terms that annotate genes in a network are found to be significantly enriched in at least one gene

cluster, leaving about one-half of the functional space unexplored (see Supplementary Table 3). For the significantly enriched GO BP terms, the functional interactions uncovered by the gene-centric and the FMM approaches are in significant agreement, with an average AUROC of 88% and all p-values $\leq 1 \times 10^{-323}$ (see Supplementary Figures 13 and 14). These results confirm that the GO BP terms that are enriched in the same gene cluster tend to be located close in the embedding space and thus, tend to have small association values in the FMM.

In conclusion, our FMM-based method is not only able to uncover the functional organization of biological functions that are identified by the gene-centric approach, but it goes beyond and characterizes the functional organization of all available GO BP terms.

1.2.3 The FMMs reveal the higher-order functional organizations of the GO BP terms in the network embedding spaces

In the previous section, we showed that our FMM better capture the pairwise functional interactions between GO BP terms than the traditional gene-centric approach. Here, we ask if the FMM can uncover the higher-order functional organization of the GO BP terms in a network embedding space. To this aim, we embed all tissue-specific PPI networks by applying our NMTF-based methodology on the PPMI matrix representations of the networks (detailed in sections 2.1 and 2.3 of the main manuscript). We generate these embedding spaces with 200 dimensions since this dimensionality corresponds to the optimal dimensionality of such spaces (as detailed in section 2.5 of the main text). Then, we apply our FMM-based method to embed and capture the relative positions of the GO BP terms in the resulting network embedding spaces (detailed in section 2.4 of the main text). To reveal the higher-order functional organization of the GO BP terms in the network embedding spaces, we apply the hierarchical clustering method Pvclust (Suzuki and Shimodaira, 2006) to the rows and columns (representing GO BP terms) of the FMMs. Pvclust evaluates the statistical significance of each cluster in the hierarchy by computing its Approximately Unbiased p-value (AU) (Suzuki and Shimodaira, 2006). Clusters with an AU value greater than or equal to 95% are considered to be strongly supported by the data, i.e., they are not expected by random.

On average over our eight tissues-specific PPI network embedding spaces, we find that about

53.62% of the clusters in the hierarchies are statistically significant with AUs greater than or equal to 95%. In detail, we find that 54%, 54%, 55%, 52%, 53%, 54%, 53%, and 54% of the clusters in the hierarchy are statistically significant with AUs greater than or equal to 95% for breast cancer, breast glandular cells, prostate cancer, prostate glandular cells, lung cancer, lung pneumocytes, colorectal cancer and colorectal glandular cells tissue-specific PPI embedding space, respectively. Importantly, these significant clusters cover all the GO BP terms that annotate the tissues-specific PPI networks. Furthermore, by reordering the rows and columns of the FMMs according to their corresponding hierarchical clusterings, we observe evident hierarchical organizations of the GO BP embedding vectors in the different network embedding spaces (see Supplementary Figures 15 and 16)

In conclusion, these results demonstrate that our FMM methodology captures the higher-order organization of the GO BP terms in the network embedding space. While these results motivate us to compare FMMs across different conditions to uncover condition-related changes in the functional organization of GO BP terms in the network embedding spaces, the extraction of novel knowledge from the hierarchical organization of the GO BP terms is a subject of future study.

1.2.4 FMM discriminates between functionally and not functionally organized embedding spaces

In section 3.1 of the main manuscript, we use our novel FMM-based method to confirm that the embedding spaces of both, cancer and control, are functionally organized. Here, we compare these results against a randomized experiment, i.e., when rewiring the previous PPI networks. In particular, for each tissue-specific PPI network, we randomly rewire the corresponding adjacency matrix and compute its corresponding PPMI matrix (detailed in section 2.1, of the paper). We follow the same protocol as used for the real tissue-specific networks to generate the corresponding "random" embedding space (detailed in section 2.3, of the paper). Next, we apply our FMM-methodology to obtain the embedding vectors of each of the GO BP annotations and the mutual positions of these vectors, which we call "distances", in the "random" embedding spaces (detailed in section 2.4, of the paper). We evaluate the functional organization of these "random" embedding

spaces by using the same clustering method as we use with the real PPI networks (detailed in section 2.6, of the paper). For each tissue-specific PPI network, we repeat this procedure 100 times. In each repetition, we statistically test if those annotations whose embedding vectors cluster together based on their mutual positions in the space have a statistically significant higher Lin's semantic similarity than those annotations whose embedding vectors do not cluster. For this test, we use the Mann-Whitney U test (keeping the corresponding p-value in each repetition). After all the repetitions are finished, we correct the p-values for multiple tests by using the Bonferroni correction (Brown, 2008). As expected, we do not find a statistically significant difference in the Lin's semantic similarity between the annotation whose embedding vectors cluster and the annotations whose embedding vectors do not cluster in the space. Hence, we conclude that the "random" embedding spaces are not functionally organized (see Supplementary Table 6). These results demonstrate that our methodology correctly discriminates between functionally and not functionally organized embedding spaces.

1.2.5 FMMs identify novel cancer-related functions

In section 3.2, of the main paper, we use our novel FMM-based methodology to predict new cancer-related functions and we verify the importance of one of our cancer-related predictions (the first annotation in our top 10 annotations predicted to be cancer-related, that we could not validate in the currently available literature). In this section, we extend this discussion for the remaining top 10 predicted cancer-related annotations. Starting with breast cancer, first we discuss the viral translational termination reinitiation. This function could be connected with the alternative transcriptional regulation pathways described in cancer (Vaklavas *et al.*, 2017). In the same cancer, we also find as predicted to be cancer-related the RNA phosphodiester bond hydrolysis, endonucleolytic. This function could be connected with the regulatory roles of RNA modifications reported in this cancer type (Kumari *et al.*, 2021). Following with prostate cancer, we find the positive regulation of endoplasmic reticulum unfolded protein response. The accumulation of unfolded protein in the ER induces this unfolded protein response as our predicted cancer-related function. It has been shown that the upregulation of this response could provide a growth advantage to tumor

cells (So *et al.*, 2009). Regarding lung cancer, we find the viral translational termination reinitiation as predicted cancer-related function. As discussed for breast cancer (see section 3.2 of the main paper), this process could also be connected with the alternative transcriptional regulation pathways described in cancer (Vaklavas *et al.*, 2017). In lung cancer, we also find the positive regulation of transcription regulatory region DNA binding as predicted cancer-related function. This processes could be connected with the well-known deregulation of the gene expression observed in different cancers (Malik and Brown, 2000). In conclusion, we demonstrate that our as predicted cancer-related function are indeed cancer-related. Thus, our novel FMM-based methodology can be used to identify new cancer-related functions.

1.2.6 Towards pan-cancer functions

In section 3.2 of the main paper, we use the total change of the distances of the annotation embedding vectors ("movement") between cancer and control embedding spaces, to identify the set of *shifted* annotations. We demonstrate that these *shifted* annotations are cancer-related and that they can be used to predict new cancer-related functions for each cancer type. In this section, we explore if there are common biological functions that are *shifted* in all four studied cancers. We hypothesize that common *shifted* annotations may represent those functions that are commonly altered in all cancers. To this end, we analyze the overlap between the *shifted* annotations of the four cancer types. We find a statistically significant intersection of eight annotations between the *shifted* functions in each cancer type (permutation test with p-value < 0.05). In particular, we randomly sample, 100 times, the equivalent number of the top-shifted functions for each cancer type, and we compute the times, n, the overlap in the randomized experiments is equal or higher than the observed (eight). Then, we derive the p-value by dividing the n times by the number of permutations (100).

To explore the meaning of these eight common annotations, we summarize them into functional domains (Supplementary section 1.1.1). We find five functional clusters: cellular response to chemokine (GO:1990869 and GO:0008543), histone phosphorylation (GO:0016572), positive regulation of the RNA export from nucleus (GO:0046833), response to radiation (GO:0009314 and

GO:0006970), and stress-activated MAPK cascade (GO:0051403 and GO:0007254). To confirm the link between these five clusters and cancer, we explore the literature that studies these domains. As expected, we find that their link to cancer is coherent. For instance, dysregulation of the MAPK signaling cascades is known to be involved in the progression of various human cancers (Rezatabar *et al.*, 2019). Regarding the response to radiation, healthy cells are known to react to radiation in three ways: arrest cell cycle progression, repair DNA lesions, or apoptosis (Li *et al.*, 2001). In cancer cells, these three reactions are known to be deregulated (Sharma *et al.*, 2019). In the case of the histone phosphorylation and RNA export from the nucleus, it could be related to the epigenetic alterations, and the dysregulation of nuclear trafficking observed in cancer (Rezatabar *et al.*, 2019; Borden, 2020). Finally, the response to chemokines has been identified to play an important role in the tumor microenvironment (Vilgelm and Richmond, 2019). Altogether, these results suggest that the functions that are *shifted* in all cancers may define generic cancer-related functions that are normally deregulated in all tumors.

To further evaluate if these eight annotations represent general mechanisms of cancer, we investigate their link with the cancer hallmarks (Hanahan and Weinberg, 2011). First, we calculate the Lin's semantic similarity (Lin *et al.*, 1998) of our eight common *shifted* annotations with the set of GO BP terms that are defined as the hallmarks of cancer by Chen *et al.* (2021). We consider an annotation to be related to one of the cancer hallmarks if it has at least a Lin's semantic similarity of 0.7 with one of the annotations that are included in the identified set of GO BP terms related to the cancer hallmarks by Chen et al., 2021. We choose a semantic similarity of 0.7 since it is the threshold that other tools apply to cluster functional annotations based on their semantic similarity, e.g., REVIGO (Supek *et al.*, 2011). Interestingly, we find that our eight common *shifted* annotations are semantically similar to the following hallmarks: inducing angiogenesis, deregulation of cellular energetic, and sustaining proliferative signal (see Supplementary Figure 12). In particular, we find that the stress-activated MAPK cascade is involved in sustaining the proliferative signal. Interestingly, this classification is coherent since these signaling cascades are known to participate in cell growth and cancer proliferation (Rezatabar *et al.*, 2019; Feitelson *et al.*, 2015). Also we see that response to chemokines is semantically similar to the inducing angiogenesis hallmark. Again, this

makes sense, since chemokines are known to play an important role in the tumor microenvironment, in which they can contribute to tumor progression by inducing angiogenesis (Fousek *et al.*, 2021). Finally, we observe that response to radiation and histone phosphorylation are both semantically similar to the deregulation of cellular energetic hallmark. Alteration of the cellular epigenetic patterns has been connected with the cellular metabolism, while the response to radiation has recently been linked to the carbon metabolism of the cell (Korimerla and Wahl, 2022).

In conclusion, these results suggest that our analysis could be extended to more cancers to uncover pan-cancer functions.

1.3 Supplementary Figures

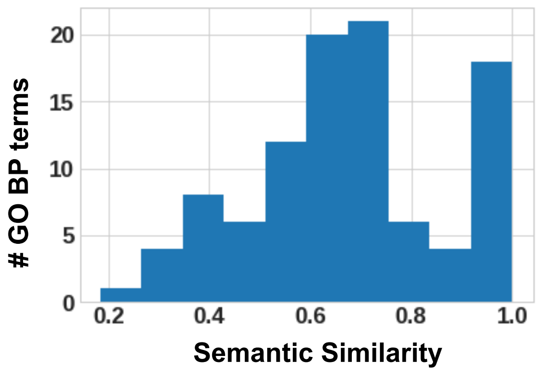

Supplementary Figure 1: Lin's semantic similarity between our set of cancer-related GO BP terms (104 annotations) and the set of GO BP terms classified as the set of GO BP cancer hallmark defined by Chen *et al.* (2021) (135 annotations). For each GO BP term in our set, we show its maximum Lin's semantic similarity to one annotation in the cancer hallmarks set.

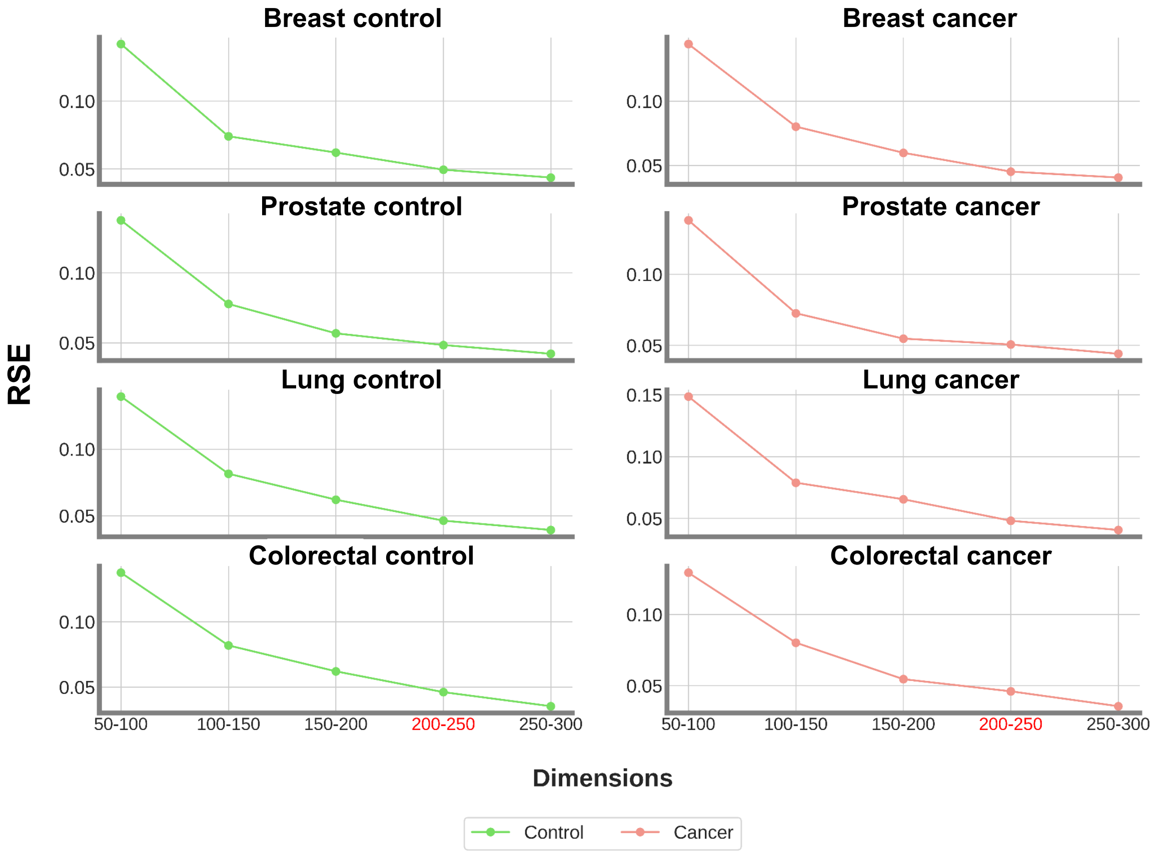

Supplementary Figure 2: For each cancer type (breast cancer, prostate cancer, lung cancer, and colorectal cancer) and its corresponding control. Each panel shows the Relative Square Error (RSE) of FMMs corresponding to the cancer and control tissues-specific embedding spaces of increasing dimensions (dimension increasing by 50 starting from 50 and ending with 300).

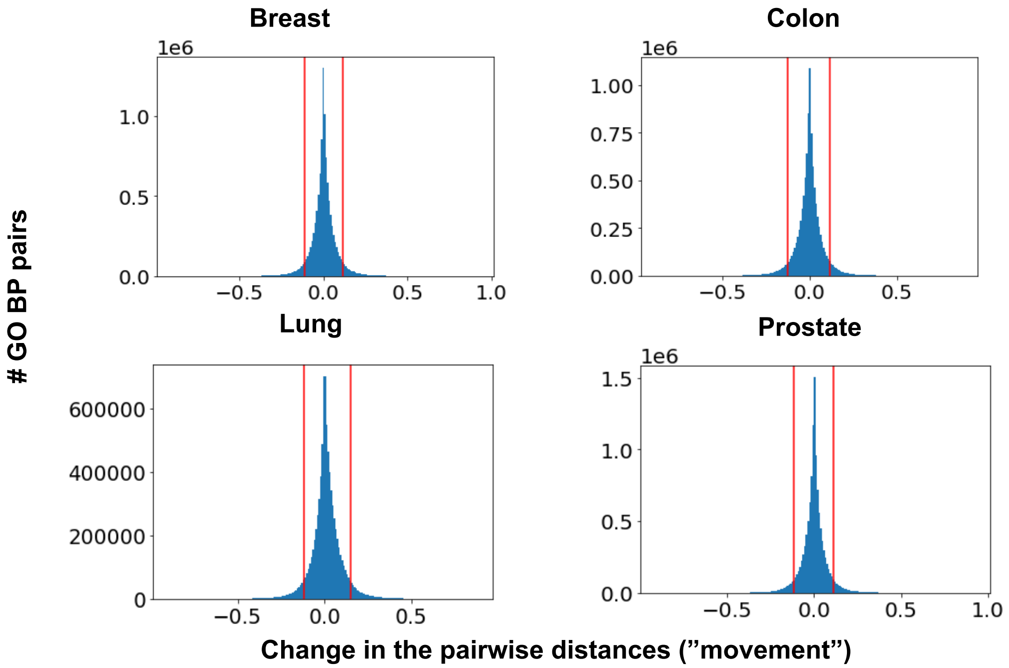

Supplementary Figure 3: Change in the pairwise distances (cosine distances), that we call “movement”, of the functional annotation embedding vectors between breast, cancer, and control embedding spaces. For a pair of annotation embedding vectors, its “movement” is the difference between the cosine distance between the two embedding vectors in one embedding space (control) and the corresponding cosine distance in the other space (cancer) (defined in section 2.7, of the paper). Thus, positive “movement” means that the two annotation embedding vectors got closer in the cancer embedding space, and negative “movement” means that the two annotation embedding vectors got further apart in the cancer embedding space. The red lines represent the 95th and 5th percentiles of the distributions. We use these thresholds to define when two annotation embedding vectors are “moving significantly apart” in the embedding space of cancer (95th percentile) or are “moving significantly closer” in the embedding space of cancer (5th percentile). The panels are for breast, lung, colon, and prostate cancers versus controls.

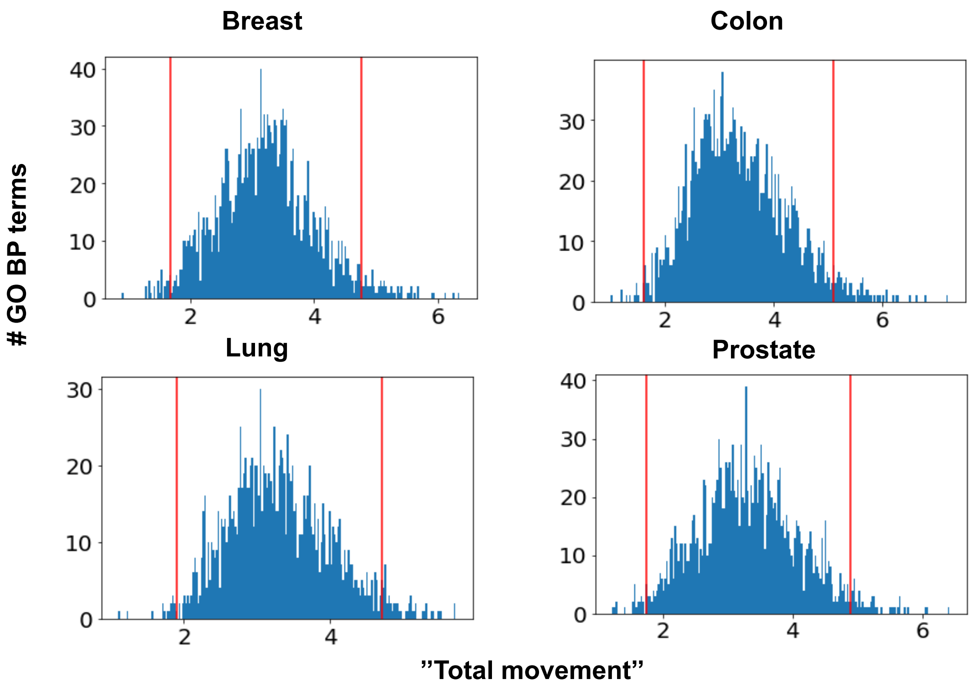

Supplementary Figure 4: "Total movement distribution" of the functional annotation embedding vectors. For each annotation embedding vector, we compute its "total movement" (defined in section 2.7, of the paper). Thus, those annotation embedding vectors that change their mutual positions, "movement", the most between control embedding space and cancer embedding space have higher "total movement" than those annotation embedding vectors that do not change their "movement". The red lines represent two standard deviations above and below the mean of the distribution. We use these thresholds to define as *shifted biological functions* those functional annotations whose embedding vectors' "total movement" is two standard deviations above the mean of the "total movement distribution." In contrast, we define as *stable biological functions* those functional annotations whose embedding vectors' "total movement" is two standard deviations below the mean of the "total movement" distribution. The distributions are for breast, lung, colon, and prostate cancers.

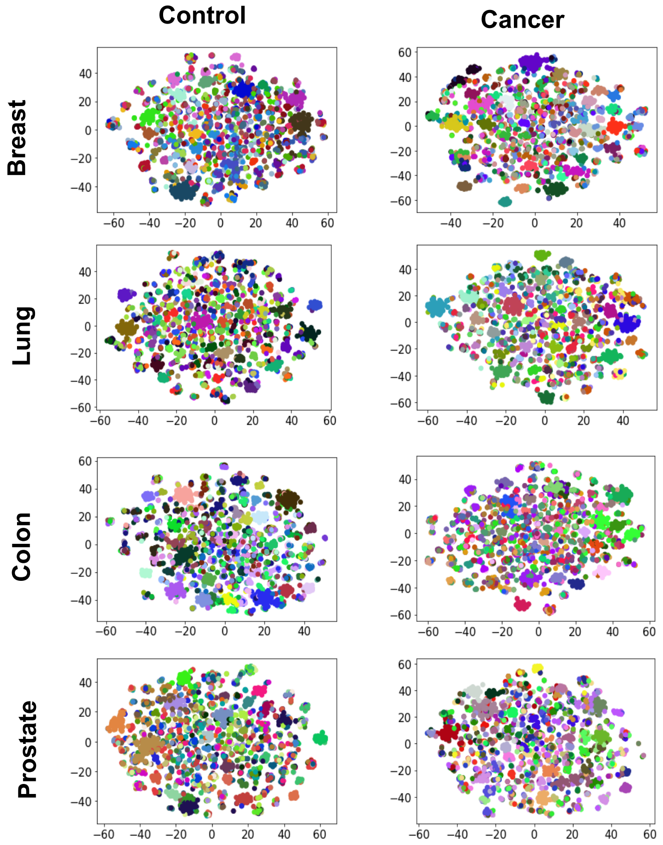

Supplementary Figure 5: The t-Distributed Stochastic Neighbor Embedding (t-SNE) of embedding vectors of the functional annotations in four cancer tissue-specific PPI embedding spaces (breast, lung, colon, and prostate) and their corresponding control tissue-specific PPI embedding spaces. For each tissue-specific PPI embedding space, we generate the embedding vectors of the functional annotations in the corresponding embedding space (detailed in section 2.4, of the paper). We use the t-SNE technique (Van der Maaten and Hinton, 2008) to visualize these embedding vectors in the tissue-specific PPI embedding space. Each dot in the plot corresponds to the embedding vector of a specific GO BP annotation. The colors of the dots correspond to the clustering of the embedding vectors of the functional annotations based on their cosine distances (detailed in section 2.6, of the paper).

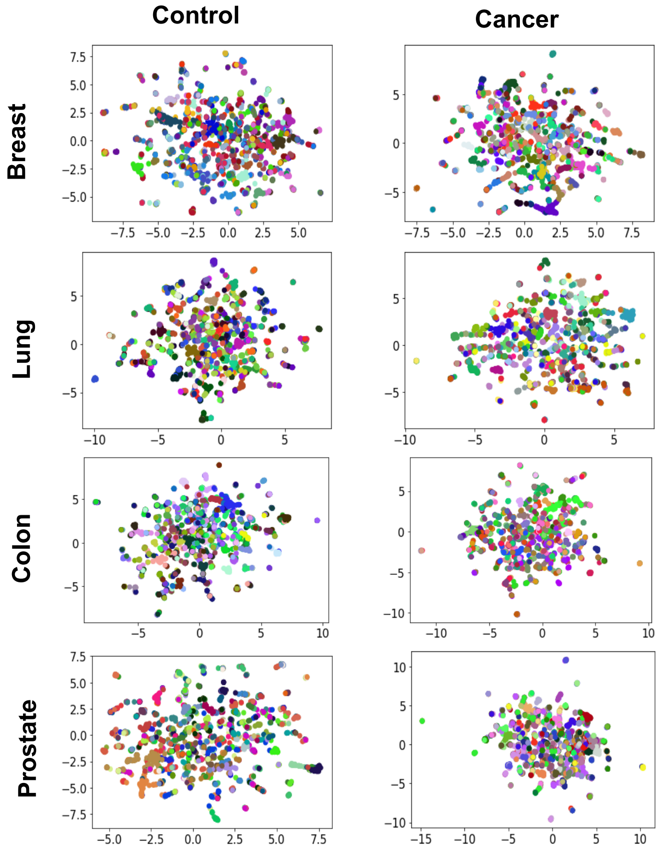

Supplementary Figure 6: The Uniform Manifold Approximation and Projection for Dimension Reduction (UMAP) of embedding vectors of the functional annotations in four cancer tissue-specific PPI embedding spaces (breast, lung, colon, and prostate) and their corresponding control tissue-specific PPI embedding spaces. For each tissue-specific PPI embedding space, we generate the embedding vectors of the functional annotations in the corresponding embedding space (detailed in section 2.4, of the paper). We use the UMAP technique (McInnes *et al.*, 2018) to visualize these embedding vectors in the tissue-specific PPI embedding space. Each dot in the plot corresponds to the embedding vector of a specific GO BP annotation. The colors of the dots correspond to the clustering of the embedding vectors of the functional annotations based on their cosine distances (detailed in section 2.6, of the paper).

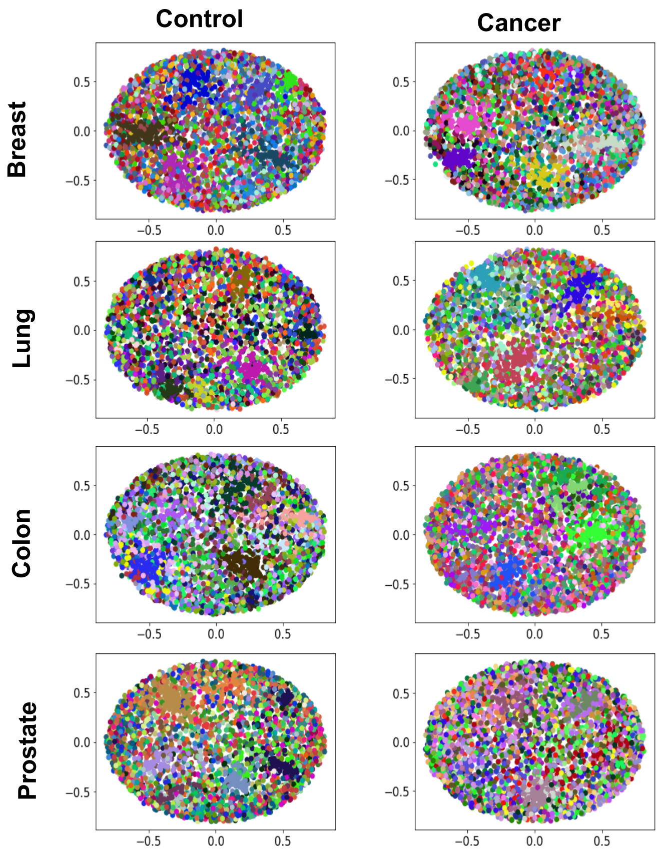

Supplementary Figure 7: The Multidimensional Scaling (MDS) of embedding vectors of the functional annotations in four cancer tissue-specific PPI embedding spaces (breast, lung, colon, and prostate) and their corresponding control tissue-specific PPI embedding spaces. For each tissue-specific PPI embedding space, we generate the embedding vectors of the functional annotations in the corresponding embedding space (detailed in section 2.4, of the paper). We use the MDS technique (Carroll and Arabie, 1998) to visualize these embedding vectors in the tissue-specific PPI embedding space. Each dot in the plot corresponds to the embedding vector of a specific GO BP annotation. The colors of the dots correspond to the clustering of the embedding vectors of the functional annotations based on their cosine distances (detailed in section 2.6, of the paper).

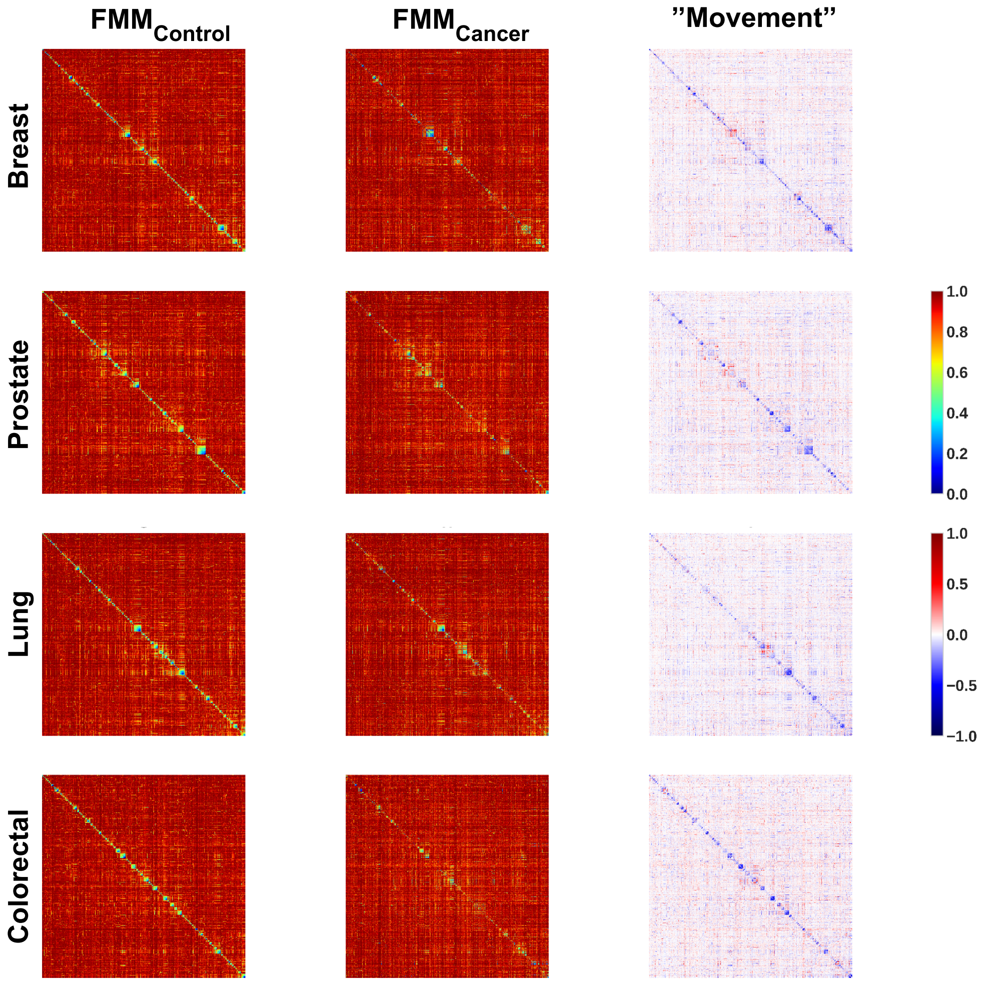

Supplementary Figure 8: The embedding vectors of GO BP terms change their mutual positions in the cancer embedding space with respect to the control embedding space, for each cancer type (breast cancer, prostate cancer, lung cancer, and colorectal cancer) and its corresponding control. Heatmaps in the first and second columns show the cosine distances (mutual positions) between the embedding vectors of the GO BP annotations in control embedding space ($FMM_{Control}$) and cancer embedding space (FMM_{Cancer}), respectively. Heatmaps in the third column show changes in the mutual positions of the embedding vectors of the functional annotations between cancer embedding space with respect to the control embedding space (computed as: $FMM_{Control}$ - FMM_{Cancer}).

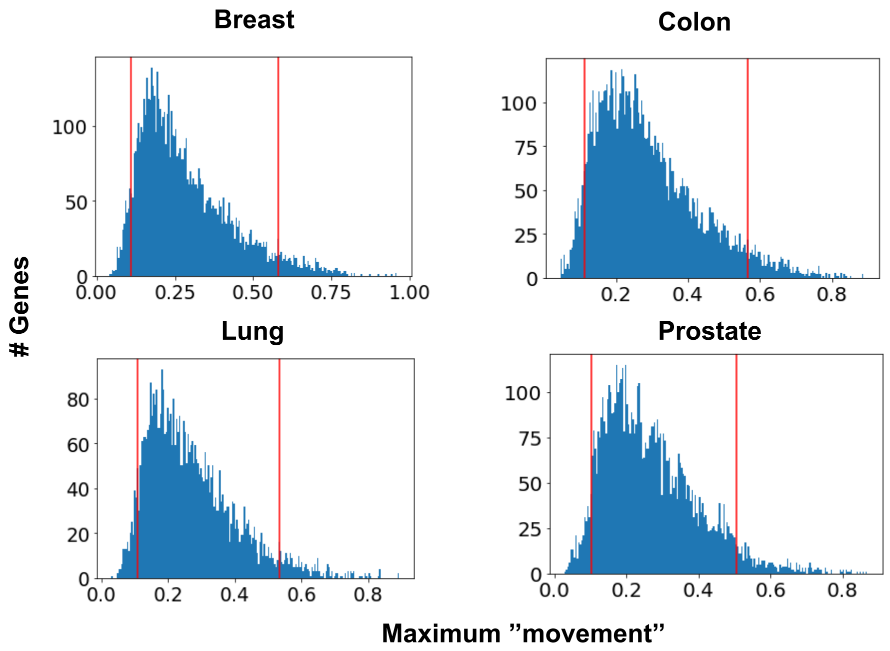

Supplementary Figure 9: Gene maximum "movement" distribution. For each gene, we have a vector with n positions, where n corresponds to the number of the "shifted" GO terms. Each entry of this n-dimensional vector corresponds to the "movement" (change of mutual positional) of the gene and the GO term. This "movement" can either be positive (a gene is going closer to the GO term in the cancer space), or negative (a gene is going further from the GO term in the cancer space). Since this "movement" is bi-directional (getting closer or further), we use the absolute value of the "movement" at each coordinate of this vector, to keep only the magnitude of this movement independently of the direction of the "movement". Then, since all the values in the n-dimensional vector are now positive, for each gene we assign as its cancer-related score the maximum value (maximum magnitude of movement) in its corresponding vector. The red lines represent the 95th and 5th percentiles of the distributions. Based on these thresholds, we consider cancer-related gene predictions whose genes that are above the 95th percentile of the maximum "movement" distribution. The distributions are for breast, lung, colon, and prostate cancers.

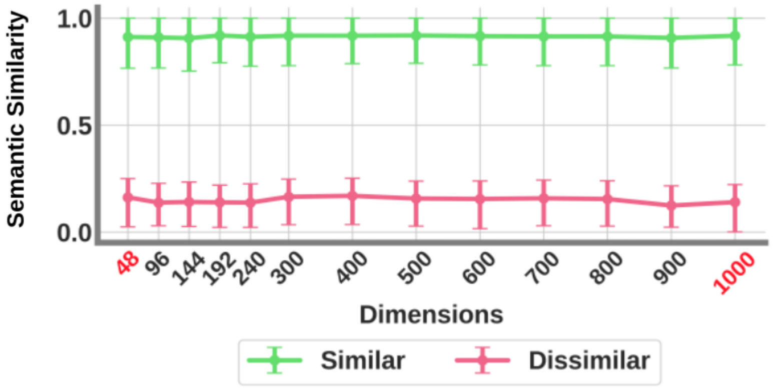

Supplementary Figure 10: For the human embedding spaces. The plot illustrates the Lin's semantic similarity between the top 500 closest functional annotation embedding vectors in the tissues-specific embedding spaces and the Lin's semantic similarity between the top 500 farthest functional annotation embedding vectors in the tissues-specific embedding spaces. The plot shows this measure for human embedding spaces generated by applying the NMTF algorithm on the corresponding tissue-specific PPI network with a different number of dimensions (48, 96, 144, 192, 240, 288, 300, 400, 500, 600, 700, 800, 900, 1000). In all the cases, we find that the Lin's semantic similarity of the 500 closest pairs of annotation embedding vectors in the embedding space is statistically higher than the average Lin's semantic similarity of the 500 farthest pairs (one-sided Mann Whitney U test p-value < 0.05).

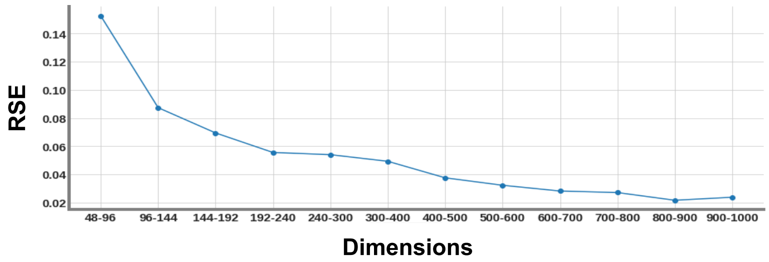

Supplementary Figure 11: For the human species-specific embedding space. Each panel shows the Relative Square Error (RSE) of FMMs corresponding to the cancer and control tissues-specific embedding spaces of increasing dimensions (from 48 to 288 with a step of 48 dimensions and from 400 to 1000 with a step of 100 dimensions).

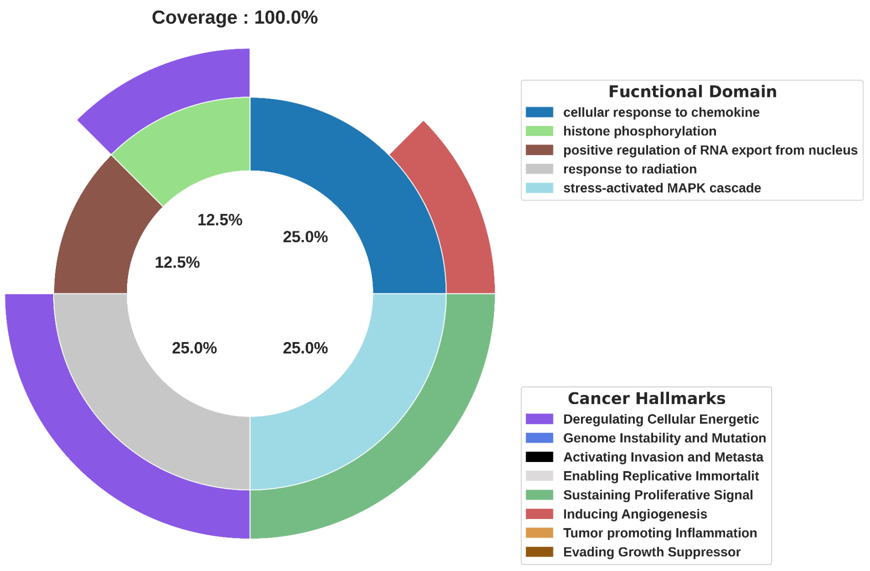

Supplementary Figure 12: We summarize the meaning of the 8 Pan-cancer annotations into functional domains (see Supplementary section 1.1.1). The panel represents the functional domains that cluster these annotations (the inner circle), and the outer represents the classification of the domains into the hallmarks of cancer.

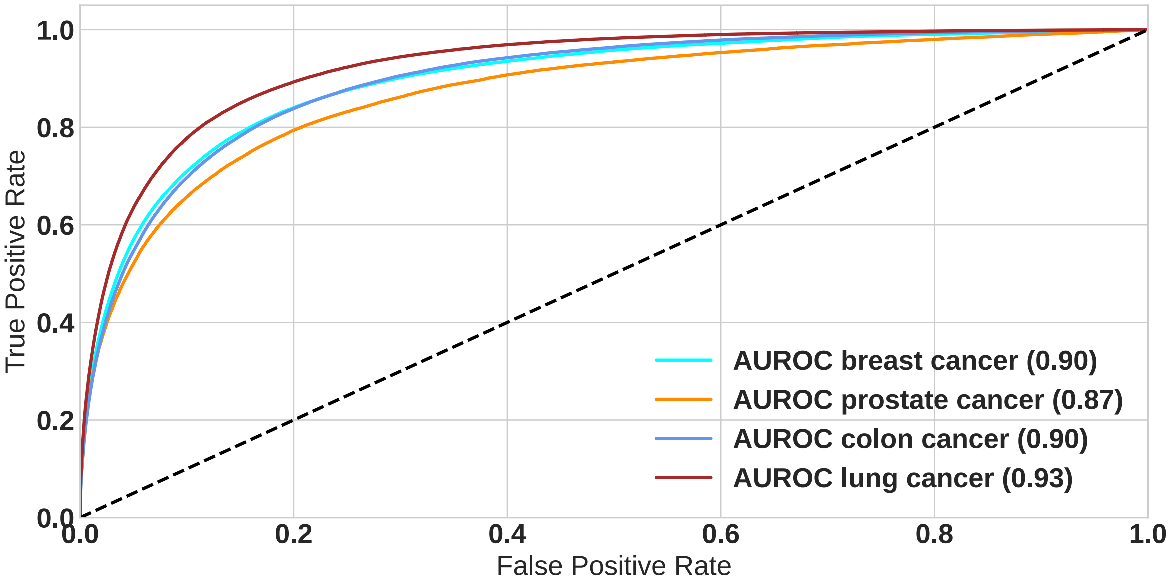

Supplementary Figure 13: Our FMM-based method uncovers the functional interactions between GO BP terms that are identified by the standard gene-centric approach (based on clustering and functional enrichment analyses) in four cancer tissue-specific PPI embedding spaces (breast, lung, colon, and prostate). For each cancer tissue-specific PPI embedding space, we take the subset of GO BP terms that are statistically enriched based on the gene-centric approach (detailed in Supplementary section 1.2.2). Then, for a pair of GO BP terms, we set the ground truth as one if they are enriched in the same cluster (zero otherwise). For the same pair, we set the prediction score as the value of their embedding vectors' cosine distance in the embedding space, as captured by the FMM. Finally, we compute the area under the receiver operating characteristic curve (AUROC) (Bradley, 1997) between the ground truth and the prediction score. Each panel shows the corresponding ROC curves with its AUROC.

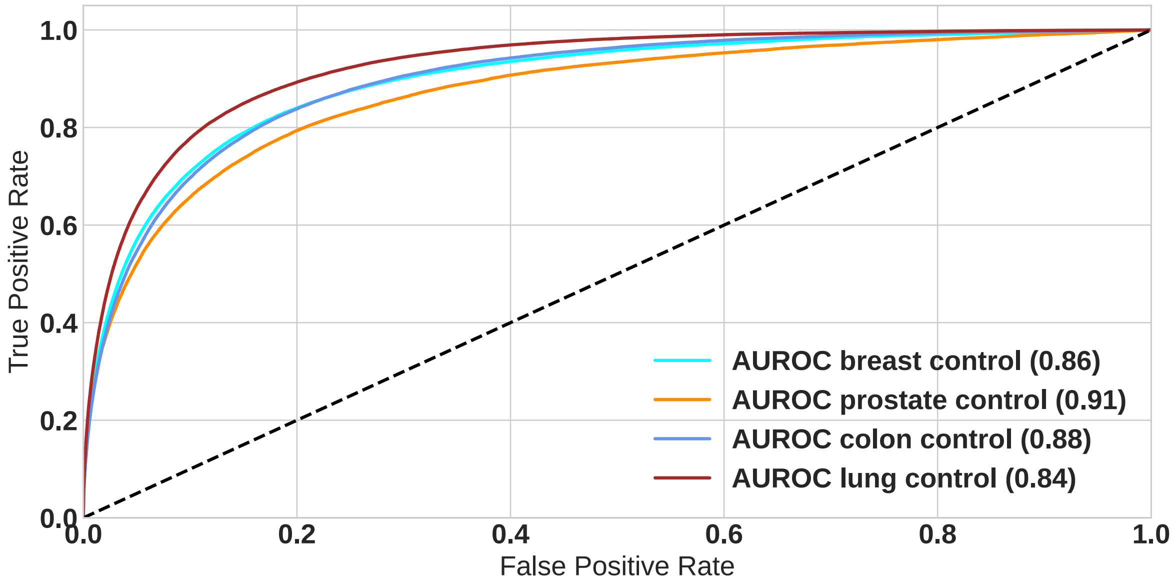

Supplementary Figure 14: Our FMM-based method uncovers the functional interactions between GO BP terms that are identified by the standard gene-centric approach (based on clustering and functional enrichment analyses) in the control tissue-specific PPI embedding spaces of four cancer types (breast, lung, colon, and prostate). For each control tissue-specific PPI embedding space, we take the subset of GO BP terms that are statistically enriched based on the gene-centric approach (detailed in Supplementary section 1.2.2). Then, for a pair of GO BP terms, we set the ground truth as one if they are enriched in the same cluster (zero otherwise). For the same pair, we set the prediction score as the value of their embedding vectors' cosine distance in the embedding space, as captured by the FMM. Finally, we compute the area under the receiver operating characteristic curve (AUROC) (Bradley, 1997) between the ground truth and the prediction score. Each panel shows the corresponding ROC curves with its AUROC.

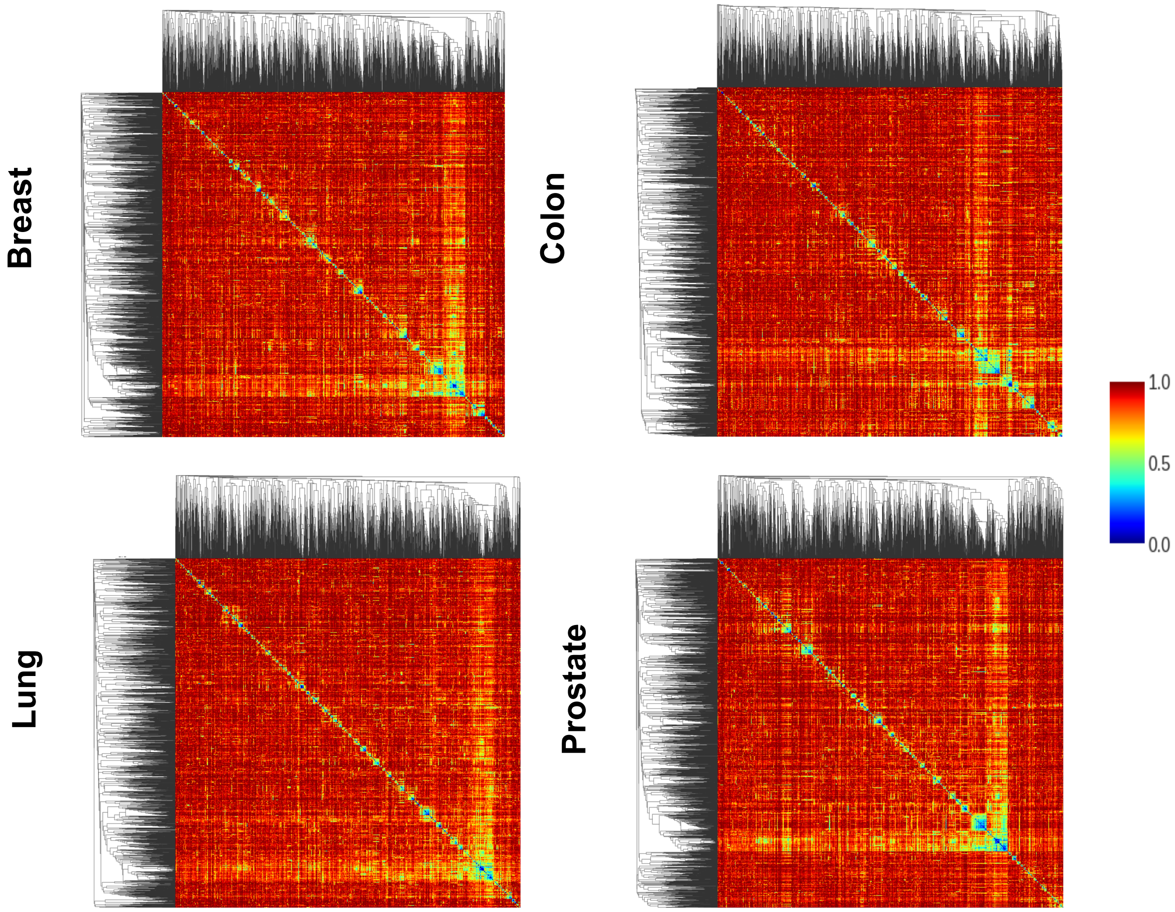

Supplementary Figure 15: Heatmaps of the FMMs of breast, lung, colon and prostate cancer tissues-specific PPI embedding spaces. For each FMM, we reorder it based on the hierarchical clustering obtained by Pvclust (see detailed in Supplementary 1.2.3). For completeness, we plot on the left and the top of each FMM heatmap the dendrogram tree of the corresponding hierarchical clustering.

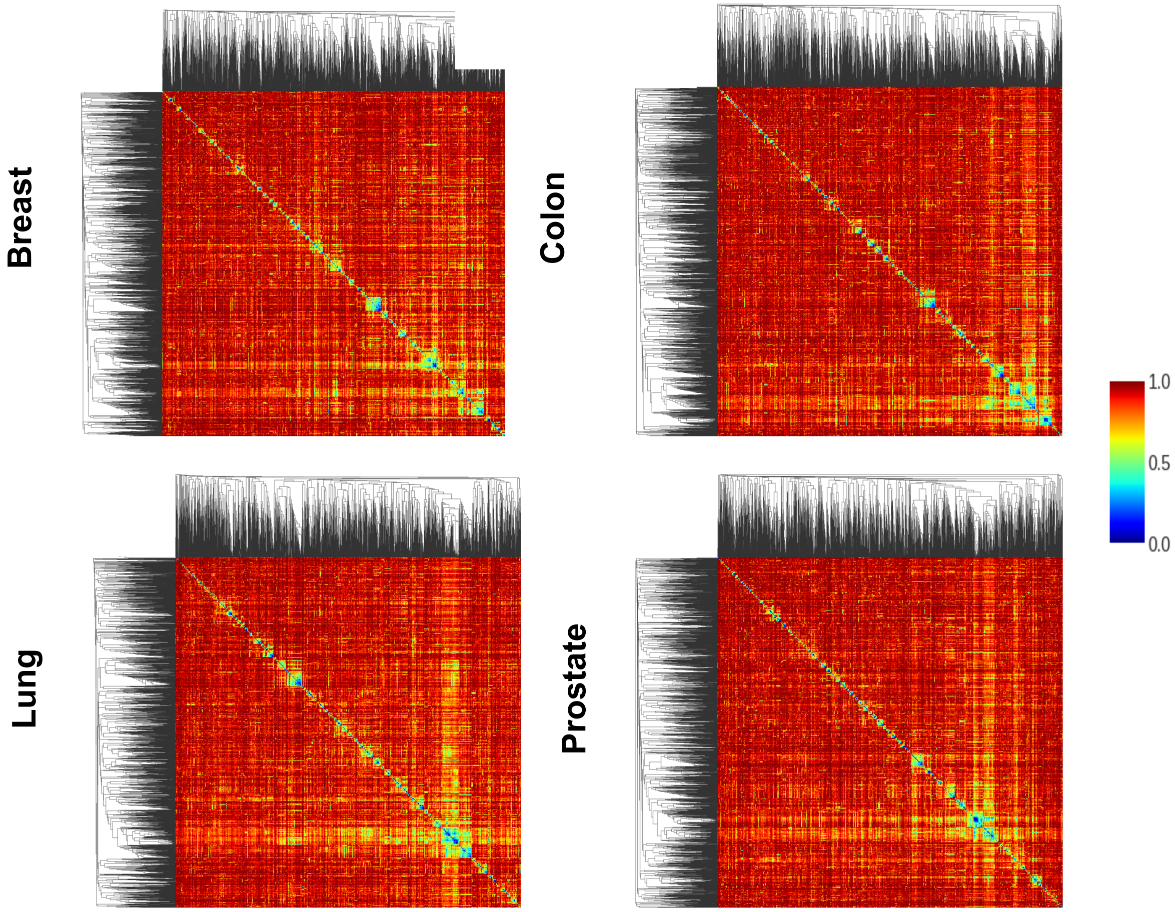

Supplementary Figure 16: Heatmaps of the FMMs of the control tissue-specific PPI embedding spaces of four cancer types (breast, lung, colon, and prostate). For each FMM, we reorder it based on the hierarchical clustering obtained by Pvclust (see detailed in Supplementary 1.2.3). For completeness, we plot on the left and the top of each FMM heatmap the dendrogram tree of the corresponding hierarchical clustering.

1.4 Supplementary Tables

Cancer	TCGA Project	# of patient samples	Disease Type
Breast	BRCA	1,098	1,095 neoplasms<br>3 adenocarcinomas
Prostate	PRAD	467	459 adenocarcinomas<br>8 neoplasms
Lung	LUAD, LUSC	1,062	533 neoplasms<br>529 adenocarcinomas
Colorectal	COAD, READ	456	389 adenocarcinomas<br>63 neoplasms

Supplementary Table 1: The statistics for the tissue-specific PPI networks in this study. Column one, "Cancer," specifies the type of cancer that we analyzed; column two, "TCGA Project," gives the name of the project from TCGA that produced the data that we used; column three, "# of patient samples" specifies the number of patient samples in the project from column two; column four, "Disease Type," specifies the numbers of patient samples from the corresponding project with a specific cancer type.

Network	#Nodes	#Edges	#Density
Breast cancer	8,498	163,893	0.45
Breast control	7,999	160,520	0.50
Prostate cancer	7,885	137,701	0.44
Prostate control	7,837	148,797	0.48
Lung cancer	7,031	126,744	0.51
Lung control	5,912	95,774	0.54
Colorectal cancer	8,941	175,081	0.43
Colorectal control	8,974	185,342	0.46

Supplementary Table 2: The statistics for the tissue-specific PPI networks in this study. Column "Network" presents the tissue-specific PPI network that we analyzed column; column, "# Nodes," presents the number of nodes in the PPI network; column, "# Edges," presents the number of edges between the nodes; column, "#Density," presents the edge density of the corresponding PPI network.

Matrix	Data set	%Clusters	%Genes	%GO
PPMI	breast cancer	81.00	23.12	52.44
PPMI	breast control	68.25	22.69	51.73
PPMI	prostate cancer	76.19	23.28	49.36
PPMI	prostate control	80.95	25.37	52.28
PPMI	lung cancer	73.13	25.28	53.01
PPMI	lung control	79.10	24.33	55.97
PPMI	colorectal cancer	77.97	22.05	49.2
PPMI	colorectal control	62.96	16.89	44.86
Adj	breast cancer	70.77	17.87	36.24
Adj	breast control	76.19	18.07	41.84
Adj	prostate cancer	77.78	14.25	40.83
Adj	prostate control	77.19	16.96	38.33
Adj	lung cancer	74.62	20.89	38.76
Adj	lung control	79.10	17.89	41.70
Adj	colorectal cancer	57.63	13.08	30.96
Adj	colorectal control	57.41	10.88	31.87

Supplementary Table 3: The embedding spaces of the most prevalent cancers (breast, prostate, lung, and colorectal cancer) and their control tissues (breast glandular cells, prostate glandular cells, lung pneumocytes, and colorectal glandular cells) are functionally organized according to the mutual positions (cosine distances) of the gene embedding vectors in the embedding space (gene perspective). For each tissue-specific PPI embedding space, we cluster genes whose embedding vectors are close in the space based on their cosine distance, and then we measure the enrichment of those clusters in GO BP annotations. The first column, "Matrix," indicates the matrix representation of the tissue-specific PPI network. The second column, "Data set," specifies the tissue-specific PPI network. The third column, "%Clusters," shows the percentage of clusters with at least one GO BP term enriched. The fourth column, "%Genes," presents the percentage of enriched genes in the clusters (out of the total number of genes in the corresponding tissue-specific PPI network). The sixth column, "%GO," shows the percentage of GO BP terms enriched in the clusters (out of the total GO BP terms that annotate the genes of the corresponding tissue-specific PPI network).

Embedding	Intra-SS	Inter-SS	Fold	p-value Fold	p-value (PPMI)
Control breast	0.18	0.16	1.10	0.0001	0.004
Cancer breast	0.18	0.16	1.10	0.0004	1.31×10^{-8}
Control prostate	0.18	0.17	1.08	0.0074	0.0004
Cancer prostate	0.18	0.17	1.08	0.0002	8.05×10^{-38}
Control colon	0.18	0.16	1.11	0.0004	0.0008
Cancer colon	0.18	0.16	1.10	0.0004	5.00×10^{-42}
Control lung	0.18	0.17	1.06	0.0020	2.53×10^{-71}
Cancer lung	0.18	0.17	1.09	0.0020	9.73×10^{-57}

Supplementary Table 4: The adjacency embedding spaces of the most prevalent cancers (breast, prostate, lung, and colorectal cancer) and their control tissues (breast glandular cells, prostate glandular cells, lung pneumocytes, and colorectal glandular cells) are functionally organized. The first column, "Embedding," lists the tissues. The second column, "Intra-SS," shows the average Lin's semantic similarity of those annotations whose embedding vectors cluster together based on their cosine distances in the embedding space. The third column, "Inter-SS," shows the average Lin's semantic similarity of those annotations whose embedding vectors do not cluster together based on their cosine distances in the embedding space. The fourth column, "Fold," displays how many times the average Lin's semantic similarity of those annotations whose embedding vectors cluster together based on their cosine distances in the embedding space is higher than of those annotations whose embedding vectors do not cluster together. The fifth column, "p-value Fold," shows the p-value from a one-sided Mann-Whitney U test comparing Lin's semantic similarity between annotations whose embedding vectors cluster together and those with non-clustered embedding vectors. The sixth column, "p-value (PPMI)," shows the p-value from a one-sided Mann-Whitney U test comparing Lin's semantic similarity between annotations that cluster together based on their proximity in the PPMI embedding space and those annotations that cluster together based on their proximity in the corresponding adjacency embedding space.

Network	#Optimal Dimensions
Breast cancer	200
Breast control	200
Prostate cancer	200
Prostate control	200
Lung cancer	200
Lung control	200
Colorectal cancer	200
Colorectal control	200
Human	240
Mouse	100
Baker's yeast	80
Fission yeast	80
Rat	100
Fruit fly	100

Supplementary Table 5: Optimal number of dimensions for each tissue-specific and species-specific PPI embedding spaces. Column "Network," specifies the tissue, or species-specific PPI network. Column, "# Optimal Dimensions," contains the optimal number of dimensions that we found experimentally as explained in section 2.5 of the paper, that we then used for generating the corresponding embedding space by our NMTF-based procedure explained in the paper.

Embedding	Intra	Inter	Fold	p-value
Random control breast	0.17	0.17	1.00	0.14
Random cancer breast	0.17	0.17	1.00	0.09
Random control prostate	0.17	0.17	1.00	0.06
Random cancer prostate	0.18	0.17	1.05	0.07
Random control colon	0.16	0.16	1.00	0.10
Random cancer colon	0.17	0.16	1.05	0.08
Random control lung	0.16	0.17	0.94	0.09
Random cancer lung	0.15	0.15	1.00	0.07

Supplementary Table 6: Our FMM-based method discriminates between functionally organized embedding spaces and those that are not. For each tissue-specific PPI network, we randomly rewire the networks (detailed in Supplementary section 1.2.4) and generate the embedding space by using the NMTF algorithm. Then, we use our new FMM-based method to evaluate the functional organization of these random PPI embedding spaces (detailed in section 2.6 of the main manuscript). The first column, "Embedding," lists the randomized tissue-specific PPI embedding spaces. The second column, "Intra," shows the average Lin's semantic similarity of those annotations whose embedding vectors cluster together based on their cosine distances in the randomized tissue-specific embedding space. The third column, "Inter," shows the average Lin's semantic similarity of those annotations whose embedding vectors do not cluster together based on their cosine distances in the randomized tissue-specific embedding space. The fourth column, "Fold," displays how many times the average "Intra" semantic similarity is higher than the "Inter" semantic similarity. The fifth column, "p-value," shows the one-sided Mann-Whitney U test p-value between the distributions of "Intra" and "Inter".

	Shifted	*Stable*
Breast	58	29
Prostate	49	26
Lung	53	15
Colorectal	68	13

Supplementary Table 7: Numbers of GO BP annotations in the *shifted* and *stable* sets in each cancer type. For the four cancer types: breast cancer (denoted by "Breast"), prostate cancer (denoted by "Prostate"), lung cancer (denoted by "Lung"), and colorectal cancer (denoted by "Colorectal"). Column, "*shifted*," presents the number of annotations in the set of *shifted* functions; column, "Stable", presents the number of annotations in the set of stable functions. The details about the definitions of *shifted* and *stable* sets can be found in section 2.7.

Annotation	#Norm	#Cancer related	#Bibliography
positive regulation of mrna binding	6.349	False	6
positive regulation of activated t cell proliferation	6.259	False	0
viral translational termination reinitiation	6.112	False	0
dna topological change	5.938	False	1
response to radiation	5.897	False	90
positive regulation of phagocytosis	5.692	False	2
establishment of mitotic spindle localization	5.679	False	139
regulation of lipid kinase activity	5.637	False	4
rna phosphodiester bond hydrolysis, endonucleolytic	5.579	False	0
positive regulation of mda 5 signaling pathway	5.576	False	1
positive regulation of receptor mediated endocytosis	5.558	False	121
protein localization to nucleolus	5.530	False	56
male gonad development	5.410	False	0
histone h3 s10 phosphorylation	5.396	False	0
stress activated mapk cascade	5.392	False	0
ATP generation from poly adp d ribose	5.366	False	0
negative regulation of oxidative stress induced neuron death	5.359	False	0
positive regulation of dna binding	5.292	False	0
mrna transcription	5.267	False	54
alternative mrna splicing, via spliceosome	5.256	False	1
histone phosphorylation	5.242	False	4
single strand break repair	5.223	False	12
leukocyte migration	5.188	False	34
rna secondary structure unwinding	5.171	True	0
negative regulation of nitric oxide biosynthetic process	5.166	False	0
positive regulation of rna export from nucleus	5.144	False	0
jnk cascade	5.137	False	2
response to x ray	5.134	False	0
nuclear pore complex assembly	5.117	False	0
negative regulation of trophoblast cell migration	5.116	False	0
dna ligation involved in dna repair	5.084	False	5
focal adhesion assembly	5.073	False	5
arachidonic acid metabolic process	5.069	False	0
positive regulation of production of miRNA involved in gene silencing	5.065	False	33
trail activated apoptotic signaling pathway	5.041	False	0
positive regulation of cell death	5.034	False	32
nucleotide excision repair, dna gap filling	4.987	False	0
negative regulation of kinase activity	4.975	False	84
negative regulation of lipopolysaccharide mediated signaling pathway	4.973	False	0
positive regulation of nitric oxide biosynthetic process	4.954	False	0
regulation of cohesin loading	4.954	False	1
maintenance of protein location in mitochondrion	4.934	False	0
establishment of protein localization to mitochondrion	4.934	False	0
cellular response to sodium arsenite	4.932	False	5
negative regulation of interleukin 1 beta production	4.926	False	0
protein poly adp ribosylation	4.905	False	1
dna synthesis involved in dna repair	4.881	True	95
cellular response to amyloid beta	4.877	False	3
positive regulation of rig i signaling pathway	4.874	False	0
activation of innate immune response	4.868	False	21
regulation of protein kinase activity	4.848	False	0
amyloid fibril formation	4.834	False	1
positive regulation of neutrophil chemotaxis	4.830	False	0
negative regulation of production of mirnas involved in gene silencing by mirna	4.825	False	3
glycolytic process	4.818	False	2
regulation of mitotic cell cycle phase transition	4.799	False	126
positive regulation of substrate adhesion dependent cell spreading	4.791	False	1
response to osmotic stress	4.785	False	1

Supplementary Table 8: *Shifted* GO BP terms in breast cancer (58 GO BP terms). Column one, "Annotations," presents the *shifted* annotations in breast cancer; column two, "# Norm," presents the "total movement" of the annotations (detailed in section 2.7, of the paper); column three, "# Cancer related," presents whether the annotations is part of our cancer-related set (True) or not (False); column four, "# Bibliography," presents the number of publications in Pubmed that relate the function to breast cancer.

Annotation	#Norm	#Cancer related	#Bibliography
notch signaling pathway	6.419	False	20
negative regulation of stem cell differentiation	6.074	False	20
nucleotide binding oligomerization domain containing 2 signaling pathway	6.039	False	2
positive regulation of response to dna damage stimulus	5.795	False	218
cleavage furrow formation	5.739	False	0
positive regulation of endoplasmic reticulum unfolded protein response	5.664	False	0
interleukin 6 mediated signaling pathway	5.658	False	1
negative regulation of interleukin 1 beta production	5.642	False	13
JNK cascade	5.636	False	3
positive regulation of t cell cytokine production	5.578	False	1
histone H3k4 methylation	5.558	False	5
positive regulation of macrophage chemotaxis	5.528	False	3
smad protein complex assembly	5.490	False	4
sprouting angiogenesis	5.378	False	2
regulation of protein containing complex assembly	5.325	False	0
positive regulation of RAS protein signal transduction	5.295	False	2
amyloid fibril formation	5.275	True	0
histone H3k4 monomethylation	5.253	True	1
histone H3k4 dimethylation	5.253	True	1
positive regulation of transcription from RNA polymerase II promoter in response to stress	5.249	False	0
stress activated MAPK cascade	5.248	True	12
response to estradiol	5.228	False	921
apoptotic signaling pathway	5.219	False	194
histone succinylation	5.215	False	0
JNK cascade	5.184	False	3
cellular response to laminar fluid shear stress	5.152	True	0
fibroblast growth factor receptor signaling pathway	5.133	False	464
positive regulation of apoptotic signaling pathway	5.095	False	194
response to radiation	5.087	False	60
histone phosphorylation	5.074	False	3
positive regulation of mitotic cell cycle spindle assembly checkpoint	5.053	False	0
release of sequestered calcium ion into cytosol	5.039	False	0
regulation of cell adhesion mediated by integrin	5.035	False	0
positive regulation of receptor endocytosis	5.027	False	4
phosphatidylinositol 3 kinase activity	5.024	False	1
positive regulation of jun kinase activity	5.001	False	0
positive regulation of cell death	4.982	False	4
regulation of stress fiber assembly	4.973	False	1
response to bacterium	4.957	False	0
positive regulation of cell substrate adhesion	4.955	False	0
type i interferon signaling pathway	4.955	False	0
nuclear transcribed mrna catabolic process	4.954	False	1
positive regulation of smooth muscle cell proliferation	4.948	False	1
bmp signaling pathway	4.946	False	3
negative regulation of cell cell adhesion mediated by cadherin	4.937	False	1
positive regulation of stress fiber assembly	4.933	False	0
cellular response to nicotine	4.929	False	0
negative regulation of myosin light chain phosphatase activity	4.925	False	0
insulin like growth factor receptor signaling pathway	4.898	False	0

Supplementary Table 9: *Shifted* GO BP terms in prostate cancer (58 GO BP terms). Column one, "Annotations," presents the *shifted* annotations in prostate cancer; column two, "# Norm," presents the "total movement" of the annotations (detailed in section 2.7, of the paper); column three, "# Cancer related," presents whether the annotations is part of our cancer-related set (True) or not (False); column four, "# Bibliography," presents the number of publications in Pubmed that relate the function to prostate cancer.

Annotation	#Norm	#Cancer related	#Bibliography
response to uv	5.721	False	3
transcription coupled nucleotide excision repair	5.719	False	4
positive regulation of endodeoxyribonuclease activity	5.527	False	0
cellular response to cytokine stimulus	5.493	False	1
nucleotide excision repair, dna incision	5.481	True	1,203
response to epidermal growth factor	5.407	False	3,712
viral translational termination reinitiation	5.397	False	0
positive regulation of transcription regulatory region dna binding	5.321	False	0
jnk cascade	5.315	False	1
stress activated mapk cascade	5.301	True	0
nucleotide excision repair	5.260	False	223
positive regulation of interleukin 12 production	5.183	False	0
positive regulation of rna export from nucleus	5.169	False	0
cellular response to virus	5.158	False	1
negative regulation of myeloid cell differentiation	5.129	False	1
dna replication	5.120	False	313
positive regulation of dna directed dna polymerase activity	5.115	False	0
positive regulation of activated t cell proliferation	5.102	False	0
base excision repair	5.079	False	155
cellular response to lipopolysaccharide	5.078	True	58
mismatch repair	5.061	False	176
nuclear pore complex assembly	5.0302	False	0
negative regulation of vascular associated smooth muscle cell proliferation	4.9665	False	0
DNA ADP ribosylation	4.939	False	1
protein poly ADP ribosylation	4.928	False	1
protein ADP ribosylation	4.922	False	1
positive regulation of histone phosphorylation	4.906	False	0
positive regulation of ERAD pathway	4.897	False	0
positive regulation of telomere maintenance	4.874	False	31
activation of protein kinase activity	4.870	False	6
telomere maintenance	4.864	False	31
vascular endothelial growth factor receptor signaling pathway	4.829	False	1
positive regulation of histone acetylation	4.822	False	0
apoptotic signaling pathway	4.818	False	19
cellular response to chemokine	4.816	False	0
H3k4 methylation	4.803	False	9
telomeric d loop disassembly	4.779	False	0
proteolysis involved in cellular protein catabolic process	4.777	False	0
negative regulation of cell cycle	4.771	True	1
membrane to membrane docking	4.769	False	0
positive regulation of erythrocyte differentiation	4.766	False	0
IRES dependent viral translational initiation	4.762	False	0
cellular response to exogenous dsRNA	4.761	False	0
positive regulation of interferon gamma production	4.757	False	3
positive regulation of kinase activity	4.754	True	7
DNA damage checkpoint signaling	4.749	False	4
necroptotic process	4.744	False	9
response to osmotic stress	4.734	False	0
mrna polyadenylation	4.729	False	2
torc1 signaling	4.727	False	0
negative regulation of cysteine type endopeptidase activity involved in apoptotic signaling pathway	4.725	False	0
positive regulation of translational initiation	4.719	False	0
translesion synthesis	4.718	False	17

Supplementary Table 10: *Shifted* GO BP terms in lung cancer (58 GO BP terms). Column one, "Annotations," presents the *shifted* annotations in lung cancer; column two, "# Norm," presents the "total movement" of the annotations (detailed in section 2.7, of the paper); column three, "# Cancer related," presents whether the annotations is part of our cancer-related set (True) or not (False); column four, "# Bibliography," presents the number of publications in Pubmed that relate the function to lung cancer.

Annotation	#Norm	#Cancer related	#Bibliography
DNA topological change	7.199	False	0
positive regulation of creb transcription factor activity	6.780	False	84
multicellular development	6.638	False	1
mitotic spindle midzone assembly	6.496	False	0
negative regulation of stem cell differentiation	6.276	False	14
dna adp ribosylation	6.241	False	9
response to radiation	6.186	False	30
positive regulation of dna directed dna polymerase activity	6.074	False	4
positive regulation of phosphatidylinositol 3 kinase activity	6.062	False	1
notch signaling pathway	5.995	False	43
negative regulation of interleukin 1 beta production	5.954	False	0
negative regulation of mrna splicing, via spliceosome	5.922	False	0
crd mediated mrna stabilization	5.861	False	0
protein poly adp ribosylation	5.804	False	0
cleavage furrow formation	5.754	False	0
heterotypic cell cell adhesion	5.726	False	1
positive regulation of extracellular matrix disassembly	5.719	False	0
base excision repair	5.711	False	67
positive regulation of signal transduction by p53 class mediator	5.705	False	0
viral translational termination reinitiation	5.656	False	0
positive regulation of cytokinesis	5.642	False	0
negative regulation of oxidative stress induced neuron death	5.638	False	0
establishment of mitotic spindle localization	5.628	False	0
positive regulation of snrna transcription by rna polymerase ii	5.627	False	0
cellular response to nerve growth factor stimulus	5.619	False	0
negative regulation of myosin light chain phosphatase activity	5.604	False	0
chromatin remodeling	5.599	False	46
dna damage checkpoint signaling	5.582	False	1
negative regulation of transposition	5.543	False	0
negative regulation of DNA recombination	5.535	True	0
mismatch repair	5.493	True	1,054
positive regulation of telomere maintenance	5.456	False	0
regulation of transforming growth factor beta receptor signaling pathway	5.428	True	1
desmosome assembly	5.411	False	1
regulation of phosphorylation	5.409	False	2
alternative mrna splicing, via spliceosome	5.394	False	1
positive regulation of gene expression, epigenetic	5.388	False	28
branching morphogenesis of an epithelial tube	5.381	False	0
regulation of alternative mRNA splicing, via spliceosome	5.352	False	1
positive regulation of intracellular estrogen receptor signaling pathway	5.326	False	0
IRES dependent viral translational initiation	5.320	False	3
SAMD protein complex assembly	5.315	False	0
progesterone receptor signaling pathway	5.313	False	7
mrna processing	5.304	False	11
dna replication	5.299	False	227
fibroblast growth factor receptor	5.286	True	131
translesion synthesis	5.270	False	3
prostaglandin biosynthetic process	5.261	False	0
nodal signaling pathway	5.248	False	1
positive regulation of erad pathway	5.242	False	0
ribosomal small subunit biogenesis	5.241	False	0
regulation of dna repair	5.237	False	0
positive regulation of epithelial to mesenchymal transition	5.215	True	6
positive regulation of helicase activity	5.208	False	0
stress induced premature senescence	5.198	False	3
negative regulation of ubiquitin protein ligase activity	5.191	False	0
dosage compensation by inactivation of x chromosome	5.190	False	0
endosomal vesicle fusion	5.179	False	0
response estrogen	5.178	True	62
neuropilin signaling pathway	5.158	False	1
positive regulation of mrna binding	5.157	False	0
negative regulation of glial cell apoptotic process	5.152	False	0
regulation of cell substrate adhesion	5.147	False	3
maintenance of DNA	5.136	True	1
activation of innate immune response	5.113	False	7
ERAD pathway	5.111	False	1
DNA damage checkpoint signaling	5.111	False	11
epithelial to mesenchymal transition	5.105	False	234

Supplementary Table 11: *Shifted* GO BP terms in colorectal cancer (58 GO BP terms). Column one, "Annotations," presents the *shifted* annotations in colorectal cancer; column two, "# Norm," presents the "total movement" of the annotations (detailed in section 2.7, of the paper); column three, "# Cancer related," presents whether the annotations is part of our cancer-related set (True) or not (False); column four, "# Bibliography," presents the number of publications in Pubmed that relate the function to colorectal cancer.

Sample	Avg Distance Annotate	Avg Distance Not-Annotate
Breast cancer	0.571	0.920
Breast control	0.575	0.921
Prostate cancer	0.598	0.912
Prostate control	0.576	0.926
Colorectal cancer	0.520	0.922
Colorectal control	0.514	0.908
Lung cancer	0.578	0.920
Lung control	0.593	0.922

Supplementary Table 12: The embedding vectors of the biological functions (GO BP terms) are significantly closer in space to the embedding vectors in the same space of the genes that they annotate than to the embedding vectors of other genes. Column, " Sample," presents the tissues-specific PPI networks. Column, "Avg Distance Annotate," presents the average cosine distance in the embedding space between the embedding vectors of genes and embedding vectors of those functional annotations that annotate them; column, "Avg Distance Not-Annotate," presents the average cosine distance in the embedding space between the embedding vectors of genes and embedding vectors of those embedded functional annotations that do not annotate them. In all samples, the difference between these distances is statistically significant (p-value of the Mann-Whitney U test < 0.05).

Gene name	PubMed Counts	Prognostic Marker	Pan-Cancer Marker
LDHA	87	-	cervical cancer (unfavorable), liver cancer (unfavorable), lung cancer (unfavorable)
COPG1	1	-	liver cancer (unfavorable)
RPL11	10	yes	breast cancer (favorable), renal cancer (unfavorable)
STK36	0	-	liver cancer (unfavorable)
CD86	94	-	renal cancer (unfavorable)
SMURF1	15	-	-
VRK3	0	-	renal cancer (favorable), urothelial cancer (favorable)
MAPK8IP1	2	-	renal cancer (favorable)
RPL17	1	-	liver cancer (unfavorable)
PIAS4	10	-	endometrial cancer (favorable), pancreatic cancer (favorable)

Supplementary Table 13: Top 10 *shifted* genes (the most *shifted* ones) in breast cancer. The first column, "Gene name," presents the gene names of the top 10 *shifted* genes. The second column, "PubMed Counts," presents the number of publications in Pubmed that relate the gene to breast cancer. The third column, "Prognostic Marker," indicates if the gene is a prognostic marker ("yes" if it is a marker, "-" otherwise) in breast cancer (based on survival curves collected from the Human Protein Atlas (Pontén *et al.*, 2008)); the fourth column, "Pan-Cancer Marker," presents whether the gene is a prognostic marker for other cancer types.

Gene name	PubMed Counts	Prognostic Marker	Pan-Cancer Marker
CPSF6	0	-	liver cancer (unfavorable), renal cancer (unfavorable)
PRDM11	0	-	-
SDHB	0	-	renal cancer (favorable)
GLRX2	1	-	renal cancer (unfavorable)
IFITM2	0	-	renal cancer (unfavorable)
C1orf116	0	-	renal cancer (favorable)
H2BC4	0	-	pancreatic cancer (unfavorable), renal cancer (unfavorable)
FUS	13	-	liver cancer (unfavorable)
DDX39B	0	-	renal cancer (unfavorable), urothelial cancer (favorable)
UMAD1	0	-	renal cancer (favorable)

Supplementary Table 14: Top 10 *shifted* genes in lung cancer. The first column, "Gene name," presents the gene names of the top 10 *shifted* genes. The second column, "PubMed Counts," presents the number of publications in Pubmed that relate the gene to lung cancer. The third column, "Prognostic Marker," presents if the gene is a prognostic marker ("yes" if it is a marker, "-" otherwise) in lung cancer (based on survival curves collected from the Human Protein Atlas (Pontén *et al.*, 2008)). The fourth column, "Pan-Cancer Marker," presents whether the gene is a prognostic marker for other cancer types.

Gene name	PubMed Counts	Prognostic Marker	Pan-Cancer Marker
H4C6	0	-	-
RPL11	1	-	breast cancer (favorable), renal cancer (unfavorable)
VRK3	0	-	renal cancer (favorable), urothelial cancer (favorable)
RPL17	0	-	liver cancer (unfavorable)
GGA3	0	-	endometrial cancer (unfavorable), liver cancer (unfavorable), renal cancer (unfavorable)
RPS4X	0	-	renal cancer (unfavorable), thyroid cancer (favorable)
C1orf116	0	-	renal cancer (favorable)
NAXE	1	-	endometrial cancer (unfavorable)
RARG	0	-	endometrial cancer (unfavorable), renal cancer (unfavorable)
FUS	1	-	liver cancer (unfavorable)

Supplementary Table 15: Top 10 *shifted* genes (the most *shifted* ones) in colorectal cancer. The first column, "Gene name," presents the gene names of the top 10 *shifted* genes. The second column, "PubMed Counts," presents the number of publications in Pubmed that relate the gene to colorectal cancer. The third column, "Prognostic Marker," presents if the gene is a prognostic marker ("yes" if it is a marker, "-" otherwise) in colorectal cancer (based on survival curves collected from the Human Protein Atlas (Pontén *et al.*, 2008)). The fourth column, "Pan-Cancer Marker," presents whether the gene is a prognostic marker for other cancer types.

2 Species-related application

2.1 Supplementary Materials and Methods

2.1.1 Species-specific PPI networks

Species-Specific Networks. In Supplementary section 2.2.1, we use species-specific PPI networks to validate our new FMM-based methodology. To this end, we collect the experimentally validated protein-protein interactions (PPIs) of *Homo sapiens sapiens* (human), *Saccharomyces cerevisiae* (baker's yeast), *Schizosaccharomyces pombe* (fission yeast), *Rattus norvegicus* (rat), *Drosophila melanogaster* (fruit fly) and *Mus musculus* (mouse) from BioGRID v.4.2.191 (Oughtred *et al.*, 2019). We model these species-specific PPI data as PPI networks in which nodes represent genes (or equivalently in this study, their protein products), and edges connect nodes whose corresponding proteins physically bind. The network statistics of these species-specific PPI networks are presented in Supplementary Table 18.

Network Representation. We represent the species-specific PPI networks with their positive point-wise mutual information (PPMI) matrices, X, where each entry in the matrix contains the information about how frequently two nodes co-occur in a random walk in the corresponding PPI network. Following Xenos *et al.* (2021), we use the DeepWalk closed formula by Perozzi *et al.* (2014) with its default settings, which corresponds to 10 iterations, to compute the PPMI matrix. This formula can be interpreted as a diffusion process that captures high order proximities between the nodes in the network; hence, PPMI is a richer representation than the adjacency matrix (Xenos *et al.*, 2021).

Biological Annotations. We use the Gene Ontology Biological Process (GO BP) annotations to represent the biological functions in a cell. As described in the main manuscript (section 2.1), we collect the experimentally validated genes to GO BP annotations from NCBI's web-server (collected on 28 September 2021). However, to have a more generic perspective of the functional organization of the species-specific embedding spaces, here we extend the previous set by also considering the ancestors of the annotations in the GO ontology directed acyclic graph, using

GOATOOLS (Klopfenstein *et al.*, 2018), and by following 'is_a' and 'part_of' links. Supplementary Table 19, shows the number of GO BP annotations used in each species-specific PPI network.

2.2 Supplementary Results

2.2.1 FMM captures the functional organization of different species-specific embedding spaces

In this section, we evaluate the capability of the FMM to capture biologically relevant interactions between the annotation embedding vectors, in six different species-specific embedding spaces. In particular, we take the species-specific protein-protein interaction (PPI) networks of the following species: human, baker's yeast, fission yeast, fruit fly, rat, and mouse (detailed in Supplementary section 2.1.1). We produce the embedding space of each network by using the NMTF algorithm (detailed in section 2.3, of the paper). For these embedding spaces, we find the optimal numbers of dimensions: 240, 80, 80, 100, 100, 100, and 100 for human, baker's yeast, fission yeast, fruit fly, rat, and mouse, respectively (detailed in Supplementary section 2.2.2). Next, we apply our FMM-methodology to obtain the embedding vectors of each of the GO BP annotations and the mutual positions of these vectors, which we call "distances", in the species-specific embedding spaces (detailed in section 2.4, of the paper). Having the corresponding FMMs, we explore if all six species-specific embedding spaces are functionally organized, i.e., the annotations whose embedding vectors are close in a space are more semantically similar (functionally related) than those annotations whose embedding vectors are far in the space (detailed in section 2.6, of the paper). To this end, first we calculate the Pearson's correlation coefficient between the Lin's semantic similarities of the functional annotations and the mutual positions of their embedding vectors in the space (as detailed in section 2.6, of the paper). We find negative correlations of -0.22, -0.22, -0.23, -0.16, -0.20, and -0.17 between the semantic similarities of the annotations and the distances between their embedding vectors in human, baker's yeast, fission yeast, fruit fly, rat, and mouse species-specific embedding spaces, respectively (see Supplementary Tables 16 and 17). We assess the significance of these correlations coefficients by calculating the probability of having the same results if the correlations coefficients were zero (null hypothesis).

Also, we evaluate if those annotations whose embedding vectors cluster together based on their distances in the species-specific embedding spaces, as measured by the cosine distance, are more functionally related than those annotations whose embedding vectors do not cluster in space. To

this end, we cluster the functional annotation embedding vectors based on their mutual positions (cosine distances) in the species-specific embedding spaces by applying the k-medoid algorithm to each FMM (detailed in section 2.6, of the paper). For the number of clusters, we use the heuristic rule of thumb ($k = \sqrt{\frac{n}{2}}$, where n is the number of annotations) (Kodinariya and Makwana, 2013). We end up with 59, 39, 31, 38, 43 and 56 clusters for human, baker's yeast, fission yeast, fruit fly, rat, and mouse, respectively. We observe that the annotations whose embedding vectors cluster together based on their distances in the species-specific embedding spaces, have an average Lin's semantic similarity 1.35, 1.60, 1.60, 1.41, and 1.40 times higher than those annotations whose embedding vectors do not cluster in human, baker's yeast, fission yeast, fruit fly, rat, and mouse species-specific embedding space, respectively (see Supplementary Figure 17 and 18).

To confirm the previous results, we also analyze the functional organization of random PPI networks, i.e., when rewiring the previous species-specific networks randomly. In particular, for each species-specific PPI network, we randomly rewire the corresponding adjacency matrix. Then, we follow the same protocol as we used with the real species-specific PPI networks to generate the gene embedding spaces. We obtain the FMMs of these spaces and evaluate their functional organization following the same clustering approaches explained in the previous paragraph. For each species, we repeat this procedure 100 times, calculating the average intra and inter cluster Lin's semantic similarity of the annotations and keeping the p-value of the corresponding Mann-Whitney U test. Once the 100 repetitions are finished, we correct the p-values for multiple tests by using the Bonferroni correction (Brown, 2008). As expected, we do not find a statistically significant difference in the semantic similarity between the annotations whose embedding vectors are clustered together based on their distances in the space and those annotations whose embedding vectors do not cluster in the space.

Finally, we illustrate the previous property by focusing on the annotation embedding vectors of the 500 closest and 500 farthest pairs of annotations in the species-specific embedding spaces. Although the observation remains the same (annotations whose embedding vectors are close in the embedding space have higher semantic similarity than those whose embedding vectors are distant in the space), we find a bigger difference in the Lin's semantic similarity between these sets

of annotations (see Supplementary Figure 19 and 10). Indeed, the mean average Lin's semantic similarity of the annotations corresponding to the 500 closest pairs of vectors in the embedding space is close to 0.9 in all six species-specific embedding spaces. In contrast, this average is close to 0.1 in the annotations corresponding to the 500 farthest pairs of vectors in the space. Altogether, these results demonstrate that our FMM-based method captures the functional organization of different gene embedding spaces from a functional perspective for all six species.

To conclude, we also evaluate the ability of the FMMs to capture the similarities in the functional organization of different species-specific embedding spaces (see section 2.5 of the paper). As expected, we find that the functional organization of the embedding space of evolutionary related species is more similar (lower RSE between their FMMs) than the functional organization of embedding spaces of evolutionary distant species. For instance, the RSE between human and mouse FMMs is 0.15, while it is 0.20 between human and baker's yeast (see Supplementary Tables 21 and 20). Thus, as captured by our new FMM-based method, similarities between the functional organization of different species-specific embedding spaces correctly identify the evolutionary closeness between the species. Although this observation is promising, a further exploration of its capabilities is left for future research, and we devote the rest of the paper to cancer-related applications.

2.2.2 The similarity between the FMMs of different dimensional spaces reveal the optimal dimensionality of the embedding space

In this section, we apply our FMM-based method to find the optimal dimensionality of six species-specific embedding spaces. First we produce the embedding space of each species-specific PPI network by using the NMTF algorithm (following the same methodology explained in the first paragraph of the Supplementary section 2.2.1). To generate these embeddings, we use different sets of dimensions. We use the heuristic rule of thumb ($k = \sqrt{\frac{n}{2}}$, where n is the number of nodes in each species-specific PPI network) (Kodinariya and Makwana, 2013) to define the previous set of dimensions. This heuristic rule gives 95.6, 38.3, 28.5, 47.2, 44.8, and 26.6 dimensions for human, baker's yeast, fission yeast, fruit fly, rat, and mouse, respectively. For human, we round

the dimensions to 96, and we use half of this dimensionality (48) to define the increment in the number of dimensions from 48 to 288 dimensions. For the other model organisms, we also round the dimensions to 40, 30, 50, 45, and 30 in baker's yeast, fission yeast, fruit fly, rat, and mouse, respectively. In this case we decide to choose the number of dimensions that we obtain from baker's yeast since it has the most complete PPI. Hence, similar to human, we use half of its dimensionality (20) to define the increment in the number of dimensions from 20 to 100 dimensions. As detailed in section 2.4 of the paper, we obtain the FMMs by first generating the embedding vectors of each of the GO BP annotations in each embedding space and then calculating their mutual positions. By tracking the Relative Square Errors (RSEs) of the FMMs across previous sets of dimensions (detailed in section 2.5, of the paper), we find that the mutual positions of the embedding vectors of the functional annotations converge to a stable i.e., non-changing functional organization, after 240, 80, 80, 100, 100, and 100 dimensions in human, baker's yeast, fission yeast, fruit fly, rat, and mouse embedding spaces, respectively (RSE between their FMMs plateaus, i.e., stops decreasing, see Supplementary Figures 11 and 20). We farther validate this observation by extending the sets of dimensions to 600, 700, 800, 900, 1000 dimensions in human, and 150, 200, 250, 300 in baker's yeast, fission yeast, fruit fly, rat, and mouse, respectively. As expected, we do not find an increment in the RSE after the optimal dimensionality. Thus, we conclude that 240, 80, 80, 100, 100, and 100 dimensions are optimal dimensionality for human, baker's yeast, fission yeast, fruit fly, rat, and mouse embedding spaces, respectively. We hypothesize the number of dimensions may reflect the increasing evolutionary complexity of the organisms.

2.3 Supplementary Figures

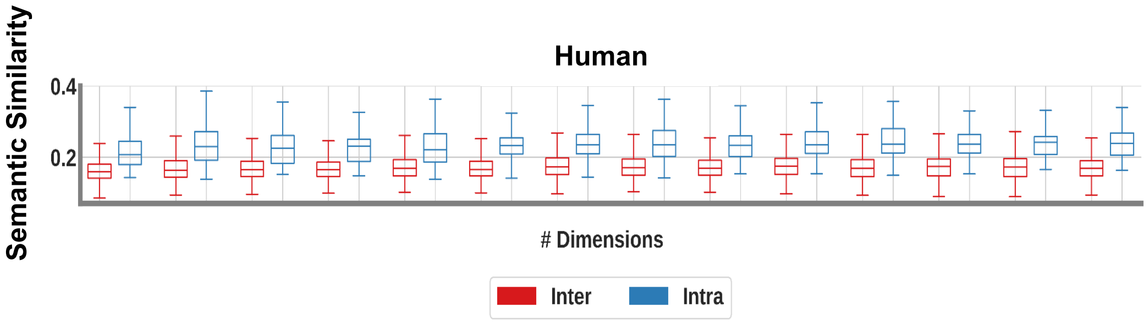

Supplementary Figure 17: Lin's semantic similarity (Semantic similarity) between the annotations whose embedding vectors are clustered together (Intra) based on mutual position in human embedding space and those that are not (Inter). The plot shows this measure for human embedding spaces generated by applying the NMTF algorithm on the corresponding tissue-specific PPI network with different number of dimensions (48, 96, 144, 192, 240, 288, 300, 400, 500, 600, 700, 800, 900, 1000). In all the cases, the intra cluster Lin's semantic similarity is statistically higher than the inter cluster one (one-sided Mann Whitney U test p-value < 0.05).

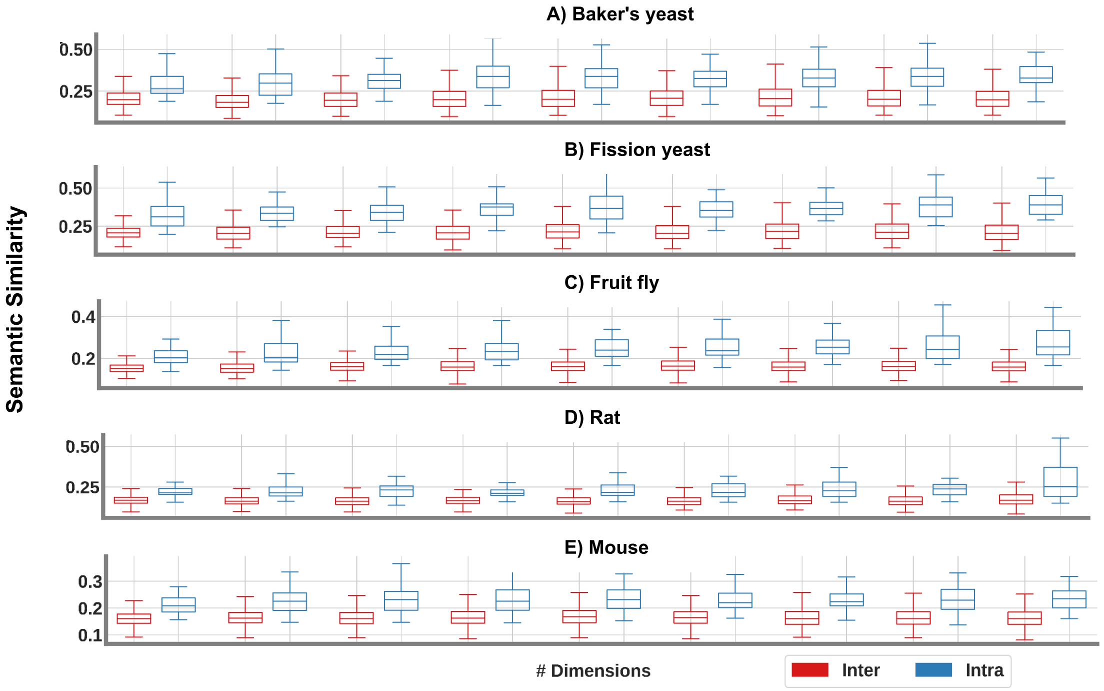

Supplementary Figure 18: For five species-specific embedding spaces: *Saccharomyces cerevisiae* (denoted by baker's yeast), *Schizosaccharomyces pombe* (denoted by fission yeast), *Rattus norvegicus* (denoted by rat), *Drosophila melanogaster* (denoted by fruit fly), and *Mus musculus* (denoted by mouse). The plot shows the Lin's semantic similarity (Semantic similarity) between the annotations whose embedding vectors are clustered together (Intra) based on mutual position in the corresponding species-specific embedding spaces and those that are not (Inter). The plot illustrate this measure for the species-specific embedding spaces generated by applying the NMTF algorithm on the corresponding tissue-specific PPI network with number of dimensions (20, 40, 60, 80, 100, 150, 200, 250 and 300). In all the cases, the intra cluster Lin's semantic similarity is statistically higher than the inter cluster one (one-sided Mann Whitney U test p-value < 0.05).

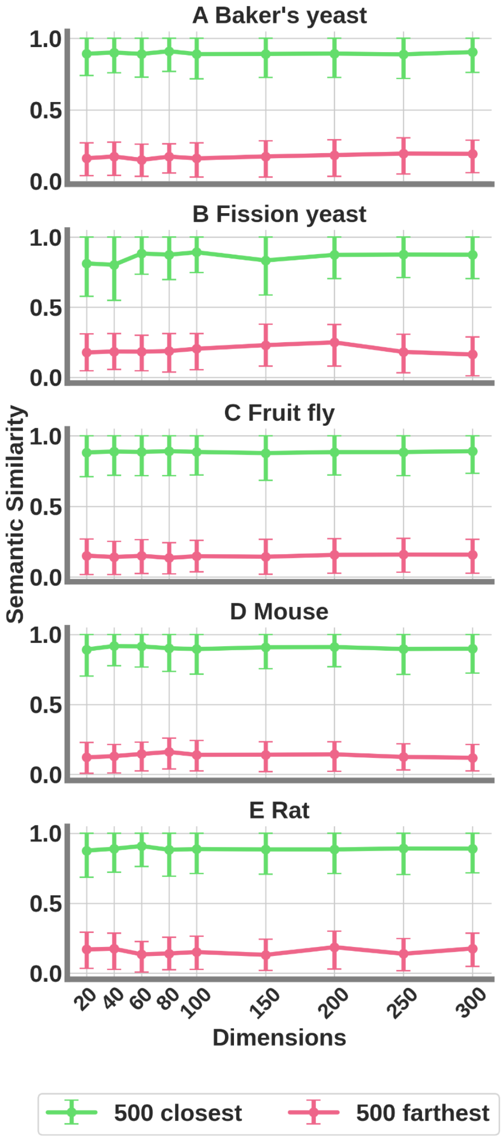

Supplementary Figure 19: For five species-specific embedding spaces: *Saccharomyces cerevisiae* (denoted by baker's yeast), *Schizosaccharomyces pombe* (denoted by fission yeast), *Rattus norvegicus* (denoted by rat), *Drosophila melanogaster* (denoted by fruit fly), and *Mus musculus* (denoted by mouse). The plot illustrates the Lin's semantic similarity between the top 500 closest functional annotation embedding vectors in the tissues-specific embedding spaces and the Lin's semantic similarity between the top 500 farthest functional annotation embedding vectors in the tissues-specific embedding spaces. The plot shows this measure for the species-specific embedding spaces generated by applying the NMTF algorithm on the corresponding tissue-specific PPI network with number of dimensions (20, 40, 60, 80, 100, 150, 200, 250 and 300). In all the cases, we find that the Lin's semantic similarity of the 500 closest pairs of annotation embedding vectors in the embedding space is statistically higher than the average Lin's semantic similarity of the 500 farthest pairs (one-sided Mann Whitney U test p-value < 0.05).

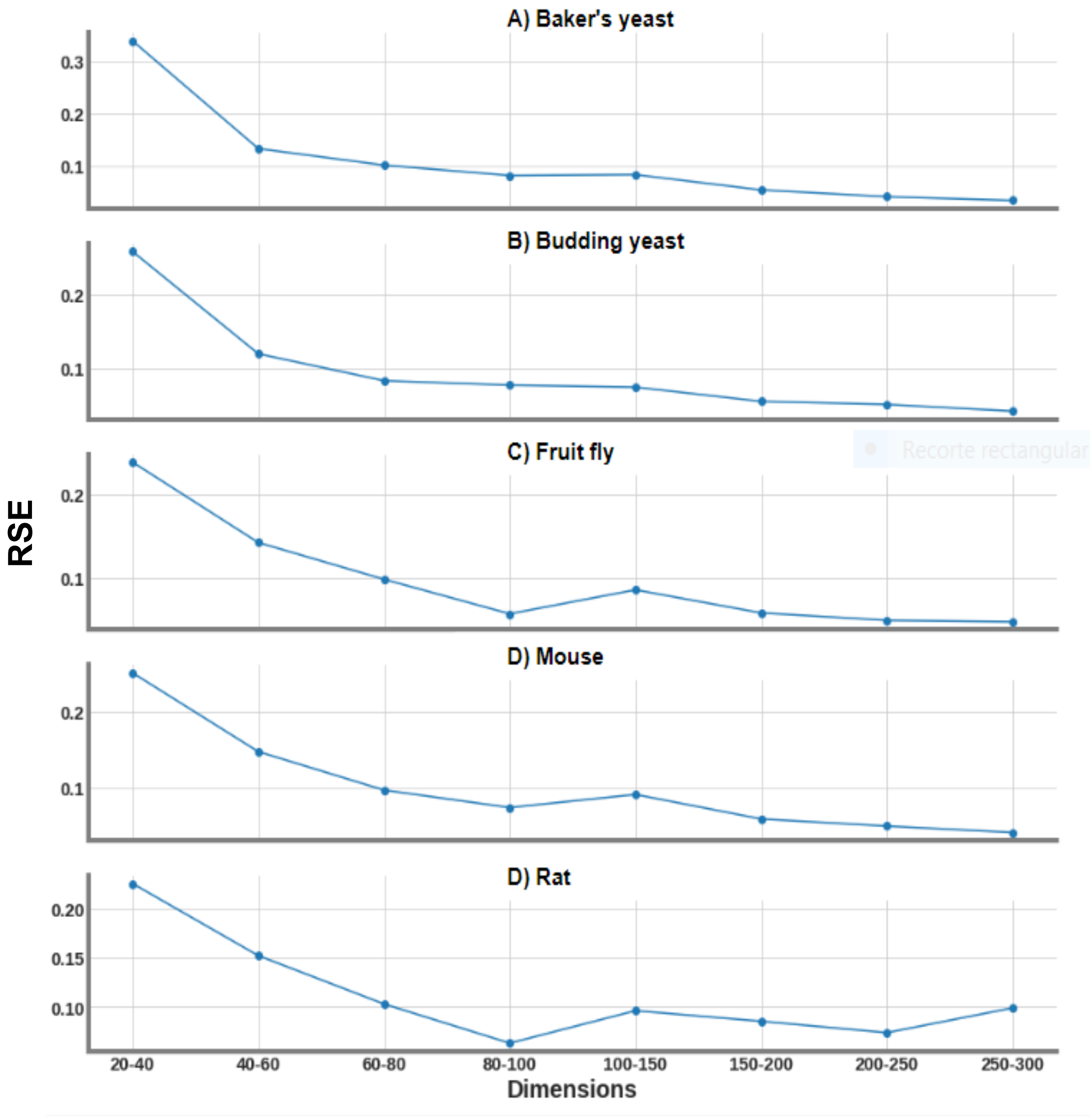

Supplementary Figure 20: For five species-specific embedding spaces: *Saccharomyces cerevisiae* (denoted by baker's yeast), *Schizosaccharomyces pombe* (denoted by fission yeast), *Rattus norvegicus* (denoted by rat), *Drosophila melanogaster* (denoted by fruit fly), and *Mus musculus* (denoted by mouse). Each panel shows the Relative Square Error (RSE) of FMMs corresponding to the cancer and control tissues-specific embedding spaces of increasing dimensions (from 20 to 100 with a step of 20 dimensions and from 100 to 300 with a step of 50 dimensions).

2.4 Supplementary Tables

Species	48-D	96-D	144-D	192-D	240-D	288-D	300-D	400-D	500-D	600-D	700-D	800-D	900-D	1000-D
Human	-0.15	-0.16	-0.17	-0.18	-0.22	-0.21	-0.21	-0.21	-0.20	-0.20	-0.21	-0.21	-0.21	-0.20

Supplementary Table 16: The first row, labeled "Species," presents each of the 13 dimensions that we tested for the human PPI network embedding spaces: 48, 96, 144, 192, 240, 288, 300, 400, 500, 600, 700, 800, 900, and 1000. Each column shows the Pearson's correlation coefficient (Benesty *et al.*, 2009) between the pairwise cosines distance of the annotations' embedding vectors in the space and the semantic similarities of the annotations (measured by the Lin's semantic similarity (Lin *et al.*, 1998)). We assess the significance of these correlations coefficients by calculating the probability of having the same results if the correlations coefficients were zero (null hypothesis). We find that all coefficients are statistically significant (p-value < 0.05). Regarding the p-value, their values are close to 0, but due to the fact that p-values in Python are float64 objects, i.e., 16 decimals are reported), they are rendered to 0.

Species	20-D	40-D	60-D	80-D	100-D	150-D	200-D	250-D	300-D
Baker's yeast	-0.19	-0.20	-0.21	-0.22	-0.22	-0.23	-0.23	-0.24	-0.25
Fission yeast	-0.20	-0.23	-0.23	-0.25	-0.27	-0.29	-0.29	-0.30	-0.30
Rat	-0.16	-0.16	-0.17	-0.17	-0.17	-0.18	-0.18	-0.19	-0.20
Fruit Fly	-0.12	-0.15	-0.15	-0.16	-0.16	-0.17	-0.18	-0.20	-0.20
Mouse	-0.17	-0.18	-0.17	-0.18	-0.18	-0.18	-0.18	-0.20	-0.20

Supplementary Table 17: Column "Species," presents species-specific PPI network embedding spaces analyzed in this study: *Saccharomyces cerevisiae* (denoted by "Baker's yeast"), *Schizosaccharomyces pombe* (denoted by "Fission yeast"), *Drosophila melanogaster* (denoted by "Fruit fly"), and *Mus musculus* (denoted by "Mouse"). Each column represents the Pearson's correlation coefficient (Benesty *et al.*, 2009) between the pairwise cosine distances of the annotations' embedding vectors in the corresponding embedding space (produced with different dimensionalities: 20, 40, 60, 80, 100, 150, 200, 250, and 300) and their Lin's semantic similarity (measured by the Lin's semantic similarity (Lin *et al.*, 1998)). We assess the significance of these correlations coefficients by calculating the probability of having the same results if the correlations coefficients were zero (null hypothesis). We find that all coefficients are statistically significant (p-value < 0.05). Regarding the p-value, their values are close to 0, but due to the fact that p-values in Python are float64 objects, i.e., 16 decimals are reported), they are rendered to 0.

Network	#Nodes	#Edges	#Density
Human	18,290	368,180	0.0022
Baker's yeast	5,887	111,307	0.0064
Fission yeast	3,269	10,958	0.0020
Fruit fly	8,917	49,756	0.0012
Mouse	8,043	26,661	0.0008
Rat	2,847	5,252	0.0013

Supplementary Table 18: The statistics of the species-specific PPI networks. For the six species: *Homo sapiens sapiens* (denoted by "Human"), *Saccharomyces cerevisiae* (denoted by "Baker's yeast"), *Schizosaccharomyces pombe* (denoted by "Fission yeast"), *Drosophila melanogaster* (denoted by "Fruit fly"), *Mus musculus* (denoted by "Mouse") and *Rattus norvegicus* (denoted by "Rat"). Column, "# Nodes", specifies the number of nodes in the species-specific PPI network; column, "# Edges," contains the number of edges between the nodes; column, "# Density," specifies the edge density of the corresponding species-specific PPI network.

Species	# GO BP terms
Human	6,864
Baker's yeast	3,042
Fission yeast	1,864
Fruit fly	3,712
Rat	2,828
Mouse	6,343

Supplementary Table 19: Number of GO BP annotations for each species-specific PPI networks. For the six species: *Homo sapiens sapiens* (denoted by "Human"), *Saccharomyces cerevisiae* (denoted by "Baker's yeast"), *Schizosaccharomyces pombe* (denoted by "Fission yeast"), *Drosophila melanogaster* (denoted by "Fruit fly"), *Rattus norvegicus* (denoted by "Rat") and *Mus musculus* (denoted by "Mouse"). Column, "# GO BP terms," presents the number of GO BP terms that annotates at least one gene in the corresponding species-specific PPI network.

	Human	Baker's yeast	Fission yeast	Fruit fly	Mouse
Human	0.000	0.204	0.228	0.182	0.159
Baker's yeast	0.204	0.000	0.157	0.178	0.217
Fission yeast	0.228	0.157	0.000	0.195	0.242
Fruit fly	0.182	0.178	0.195	0.000	0.180
Mouse	0.159	0.217	0.242	0.180	0.000

Supplementary Table 20: Pairwise relative error between the species-specific FMMs. For the five species: *Homo sapiens sapiens* (denoted by "Human"), *Saccharomyces cerevisiae* (denoted by "Baker's yeast"), *Schizosaccharomyces pombe* (denoted by "Fission yeast"), *Drosophila melanogaster* (denoted by "Fruit fly") and *Mus musculus* (denoted by "Mouse"). The table specifies the relative error between their FMMs.

	Human	Baker's yeast	Fission yeast	Fruit fly	Mouse
Human	0	529	1,017	736	89
Baker's yeast	529	0	529	1,017	1,017
Fission yeast	1,017	529	0	1,017	1,017
Fruit fly	736	1,017	1,017	0	736
Mouse	89	1,017	11,017	736	0

Supplementary Table 21: Common ancestor time, Million Yeats Ago (MYA) (O'Leary *et al.*, 2016). For the five species: *Homo sapiens sapiens* (denoted by "Human"), *Saccharomyces cerevisiae* (denoted by "Baker's yeast"), *Schizosaccharomyces pombe* (denoted by "Fission yeast"), *Drosophila melanogaster* (denoted by "Fruit fly") and *Mus musculus* (denoted by "Mouse"). The table shows the million years from the common ancestor between the species.

References

Benesty, J., *et al.* (2009). Pearson correlation coefficient. In *Noise Reduction in Speech Processing*, pages 1–4. Springer.

Benjamini, Y. *et al.* (1995). Controlling the false discovery rate: a practical and powerful approach to multiple testing. *Journal of the Royal statistical society: series B (Methodological)*, **57**(1), 289–300.

Borden, K. L. (2020). The nuclear pore complex and mrna export in cancer. *Cancers*, **13**(1), 42.

Bradley, A. P. (1997). The use of the area under the roc curve in the evaluation of machine learning algorithms. *Pattern recognition*, **30**(7), 1145–1159.

Brown, J. D. (2008). The bonferroni adjustment. *Statistics*, **12**(1).

Carroll, J. D. *et al.* (1998). Multidimensional scaling. *Measurement, judgment and decision making*, pages 179–250.

Chen, Y., *et al.* (2021). Establishing a consensus for the hallmarks of cancer based on gene ontology and pathway annotations. *BMC Bioinformatics*, **22**(1), 1–20.

Dennis, G., *et al.* (2003). David: database for annotation, visualization, and integrated discovery. *Genome Biology*, **4**(9), 1–11.

Ding, C. H., *et al.* (2008). Convex and semi-nonnegative matrix factorizations. *IEEE Transactions on Pattern Analysis and Machine Intelligence*, **32**(1), 45–55.

Feitelson, M. A., *et al.* (2015). Sustained proliferation in cancer: Mechanisms and novel therapeutic targets. In *Seminars in Cancer Biology*, volume 35, pages S25–S54. Elsevier.

Fousek, K., *et al.* (2021). Interleukin-8: A chemokine at the intersection of cancer plasticity, angiogenesis, and immune suppression. *Pharmacology & Therapeutics*, **219**, 107692.

Hanahan, D. *et al.* (2011). Hallmarks of cancer: the next generation. *Cell*, **144**(5), 646–674.

Klopfenstein, D., *et al.* (2018). Goatools: A python library for gene ontology analyses. *Scientific Reports*, **8**(1), 1–17.

Kodinariya, T. M. *et al.* (2013). Review on determining number of cluster in k-means clustering. *International Journal*, **1**(6), 90–95.

Korimerla, N. *et al.* (2022). Interactions between radiation and one-carbon metabolism. *International Journal of Molecular Sciences*, **23**(3), 1919.

Kumari, K., *et al.* (2021). Regulatory roles of rna modifications in breast cancer. *NAR Cancer*, **3**(3), zcab036.

Li, L., *et al.* (2001). Cellular responses to ionizing radiation damage. *International Journal of Radiation Oncology* Biology* Physics*, **49**(4), 1157–1162.

Lin, D. *et al.* (1998). An information-theoretic definition of similarity. In *Icml*, volume 98, pages 296–304.

Malik, K. *et al.* (2000). Epigenetic gene deregulation in cancer. *British journal of cancer*, **83**(12), 1583–1588.

McInnes, L., *et al.* (2018). Umap: Uniform manifold approximation and projection for dimension reduction. *arXiv preprint arXiv:1802.03426*.

O'Leary, N. A., *et al.* (2016). Reference sequence (RefSeq) database at NCBI: current status, taxonomic expansion, and functional annotation. *Nucleic Acids Research*, **44**(D1), D733–D745.

Oughtred, R., *et al.* (2019). The biogrid interaction database: 2019 update. *Nucleic Acids Research*, **47**(D1), D529–D541.

Park, H.-S. *et al.* (2009). A simple and fast algorithm for k-medoids clustering. *Expert systems with Applications*, **36**(2), 3336–3341.

Perozzi, B., *et al.* (2014). Deepwalk: Online learning of social representations. In *Proceedings of the 20th ACM SIGKDD International Conference on Knowledge Discovery and Data Mining*, KDD '14, pages 701–710, New York, NY, USA. ACM.

Pontén, F., *et al.* (2008). The human protein atlas—a tool for pathology. *The Journal of Pathology: A Journal of the Pathological Society of Great Britain and Ireland*, **216**(4), 387–393.

Pržulj, N. (2019). *Analyzing Network Data in Biology and Medicine: An Interdisciplinary Textbook for Biological, Medical and Computational Scientists*. Cambridge University Press.

Qiao, H. (2015). New svd based initialization strategy for non-negative matrix factorization. *Pattern Recognition Letters*, **63**, 71–77.

Rezatabar, S., *et al.* (2019). Ras/mapk signaling functions in oxidative stress, dna damage response and cancer progression. *Journal of Cellular Physiology*, **234**(9), 14951–14965.

Schlicker, A., *et al.* (2006). A new measure for functional similarity of gene products based on gene ontology. *BMC Bioinformatics*, **7**(1), 1–16.

Sharma, A., *et al.* (2019). Cancer metabolism and the evasion of apoptotic cell death. *Cancers*, **11**(8), 1144.

So, A. Y.-L., *et al.* (2009). The unfolded protein response during prostate cancer development. *Cancer and Metastasis Reviews*, **28**(1), 219–223.

Supek, F., *et al.* (2011). Revigo summarizes and visualizes long lists of gene ontology terms. *PloS One*, **6**(7), e21800.

Suzuki, R. *et al.* (2006). Pvclust: an r package for assessing the uncertainty in hierarchical clustering. *Bioinformatics*, **22**(12), 1540–1542.

Vaklavas, C., *et al.* (2017). Translational dysregulation in cancer: molecular insights and potential clinical applications in biomarker development. *Frontiers in Oncology*, **7**, 158.

Van der Maaten, L. *et al.* (2008). Visualizing data using t-sne. *Journal of machine learning research*, **9**(11).

Vilgelm, A. E. *et al.* (2019). Chemokines modulate immune surveillance in tumorigenesis, metastasis, and response to immunotherapy. *Frontiers in Immunology*, **10**, 333.

Xenos, A., *et al.* (2021). Linear functional organization of the omic embedding space. *Bioinformatics*, **37**(21), 3839–3847.
